# Integrated Analysis of the Anoikis‐Related Signature Identifies Rac Family Small GTPase 3 as a Novel Tumor‐Promoter Gene in Hepatocellular Carcinoma

**DOI:** 10.1002/mco2.70125

**Published:** 2025-03-22

**Authors:** Dong Wu, Ze‐Kun Liu, Ying Sun, Chu‐Heng Gou, Run‐Ze Shang, Meng Lu, Ren‐Yu Zhang, Hao‐Lin Wei, Can Li, Ying Shi, Cong Zhang, Yu‐Tong Wang, Ding Wei, Zhi‐Nan Chen, Huijie Bian

**Affiliations:** ^1^ Department of Cell Biology National Translational Science Center for Molecular Medicine Fourth Military Medical University Xi'an China; ^2^ State Key Laboratory of New Targets Discovery and Drug Development for Major Diseases Fourth Military Medical University Xi'an China; ^3^ Department of Hepatobiliary Surgery Xijing Hospital Fourth Military Medical University Xi'an China

**Keywords:** anoikis, hepatocellular carcinoma, prognostic signature, Rac family small GTPase 3, risk model

## Abstract

Anoikis resistance in hepatocellular carcinoma (HCC) cells boosts survival and metastasis. This study aimed to establish an anoikis‐related genes (ARGs)‐based model for predicting HCC patients’ outcomes and investigate the clinicopathological significance and function of crucial ARGs. The transcriptional expression patterns for HCC cohorts were compiled from TCGA, GEO and ICGC. Univariate and LASSO multivariate analyses were performed to screen for prognostic ARGs. Gain‐ and loss‐of‐function studies, RNA sequencing, and mass spectrometry were employed to elucidate the underlying mechanisms of ARGs in HCC. We established a five‐gene ARGs risk model for HCC prognosis, with an AUC value of 0.812 for 1‐year survival. Among the five genes, Rac family small GTPase 3 (RAC3) was upregulated in HCC relative to adjacent normal tissues and negatively correlated to overall survival and disease‐free survival of patients with HCC. Silence of RAC3 in HCC cells resulted in an increased cell apoptosis and diminished cell proliferation and invasion. Mechanistically, we uncovered that RAC3 binding with SOX6 propelled the advancement of HCC cells through NNMT‐mediated stimulation of the cAMP/MAPK/Rap1 signaling. In particular, EHop‐016, a small molecule inhibitor targeting RAC3, significantly suppressed HCC progression.

## Graphical abstract

A five‐gene risk model is constructed based on anoikis‐related genes by internal and external cross‐validation. The risk model serves as an independent prognostic factor for HCC. RAC3, a novel anoikis‐related oncogene in HCC, promotes cell proliferation and invasion, and inhibits apoptosis. RAC3 interacting with SOX6 accelerates HCC malignant phenotypes via NNMT‐mediated cAMP/MAPK/Rap1 signaling activation. Targeting RAC3 by EHop‐016 suppresses HCC progression.

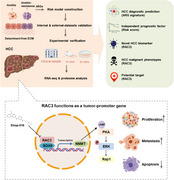

## 1 Introduction

Hepatocellular carcinoma (HCC) is recognized as one of the most frequently encountered malignancies. It is the fifth most common cancer and the second in terms of mortality in China [1]. Despite significant advancements in therapeutic strategies, including radiotherapy, surgery, and chemotherapy, the individuals diagnosed with advanced HCC still face a bleak prognosis, as evidenced by a 5‐year survival rate that hovers around 12% [2, 3]. It is of great concern that high rates of postoperative recurrence and intrahepatic/extrahepatic metastasis affect the expected outcome in individuals with HCC [4]. The occurrence of metastasis is regarded as an important event in tumor progression and remains the principal barrier to improve overall survival (OS) [5]. Resistance to anoikis is a pivotal event in the initiation and dissemination of liver cancer metastasis [6, 7]. Therefore, the development of a robust prognostic model based on anoikis‐related genes (ARGs) holds significant importance for enhancing the prediction efficiency for patients with HCC.

Anoikis is a specific type of cell death that occurs when cells detach from the extracellular matrix (ECM), which can prevent circulating tumor cells from surviving and reattaching to new matrices [8, 9]. After acquiring resistance to anoikis, malignant cells can disengage from the site of origin of the primary tumor and subsequently disseminate to other organs of the body through blood vessels or lymphatic vessels [6], thereby promoting tumor progression. Recent studies have shown that resistance to anoikis is a pivotal trait associated with the oncogenic form of epithelial–mesenchymal transition (EMT), which is a requisite for the development of metastatic cancer [8].

Multiple molecular mechanisms and pathways, including those involving cell adhesion molecules and growth factors, contribute to the modulation of resistance to anoikis [10]. It has been reported that pivotal downstream molecules, such as Src kinase, ERK1/2, Bcl‐2, Akt/PI3K, and focal adhesion kinase, play crucial roles in inhibiting apoptosis and promoting survival [8]. Some studies have indicated that CCDC178 is capable of augmenting the capacity of cells to withstand anoikis through modulating the stability of BRAP2, ultimately promoting the metastasis of HCC tumors [11]. Moreover, the protein 14‐3‐3σ is verified to enhance resistance to anoikis in HCC cells through the impeding the degradation of EGFR and modulating the stimulation of the EGFR‐mediated ERK1/2 signaling cascade [12]. Nevertheless, the molecular mechanisms by which anoikis influences the course and prognostic effects of HCC are not yet clearly understood.

Rac family small GTPase 3 (RAC3) is categorized as a small GTPase within the Rho family of proteins. It toggles between inactive GDP‐bound conformation and active GTP‐bound state, thereby orchestrating a diverse array of cellular functions, which encompass cellular growth, lipid vesicle transport, differentiation, and motility [13]. Several studies have indicated that RAC3 plays a pivotal role in facilitating the invasion and metastasis across a spectrum of malignancies, encompassing bladder cancer, breast cancer, and lung adenocarcinoma [13, 14, 15]. In addition, RAC3 may be a prognostic risk factor and has important implications for the survival and prognosis of individuals with HCC [16]. RAC3 in active GTP‐bound state acts as a co‐activator of the transcription factor ERα to induce cell proliferation and migration of breast cancer [17]. However, the mechanism by which RAC3 participates in transcriptional co‐activation in HCC remains obscure. Besides, the current understanding of the biological roles of RAC3 in HCC progression and the clinical significance of RAC3 in patients with HCC remains widely undefined.

This investigation aims to establish a defined ARGs risk model based on three datasets and systematically verified the effectiveness of model in estimating the survival for HCC patients. Furthermore, we probed the relationships between ARGs and immune subtypes and the immunotherapeutic response. In addition, experimental data from loss‐of‐function and gain‐of‐function assays indicated that enhanced expression of RAC3 promoted malignant phenotypes in HCC cells. The upregulation of RAC3 was associated with a more dismal prognosis and the expression of RAC3 protein was identified as an independent predictor in terms of OS. Moreover, we demonstrated that RAC3 interacting with SOX6 facilitated the malignant phenotypes of HCC cells via NNMT‐mediated activation of cAMP/MAPK/Rap1 signaling and inhibition of RAC3 by small molecular inhibitor EHop‐016 significantly suppressed HCC growth.

## 2 Results

### 2.1 Screening ARGs with Clinical Significance

**FIGURE 1:**
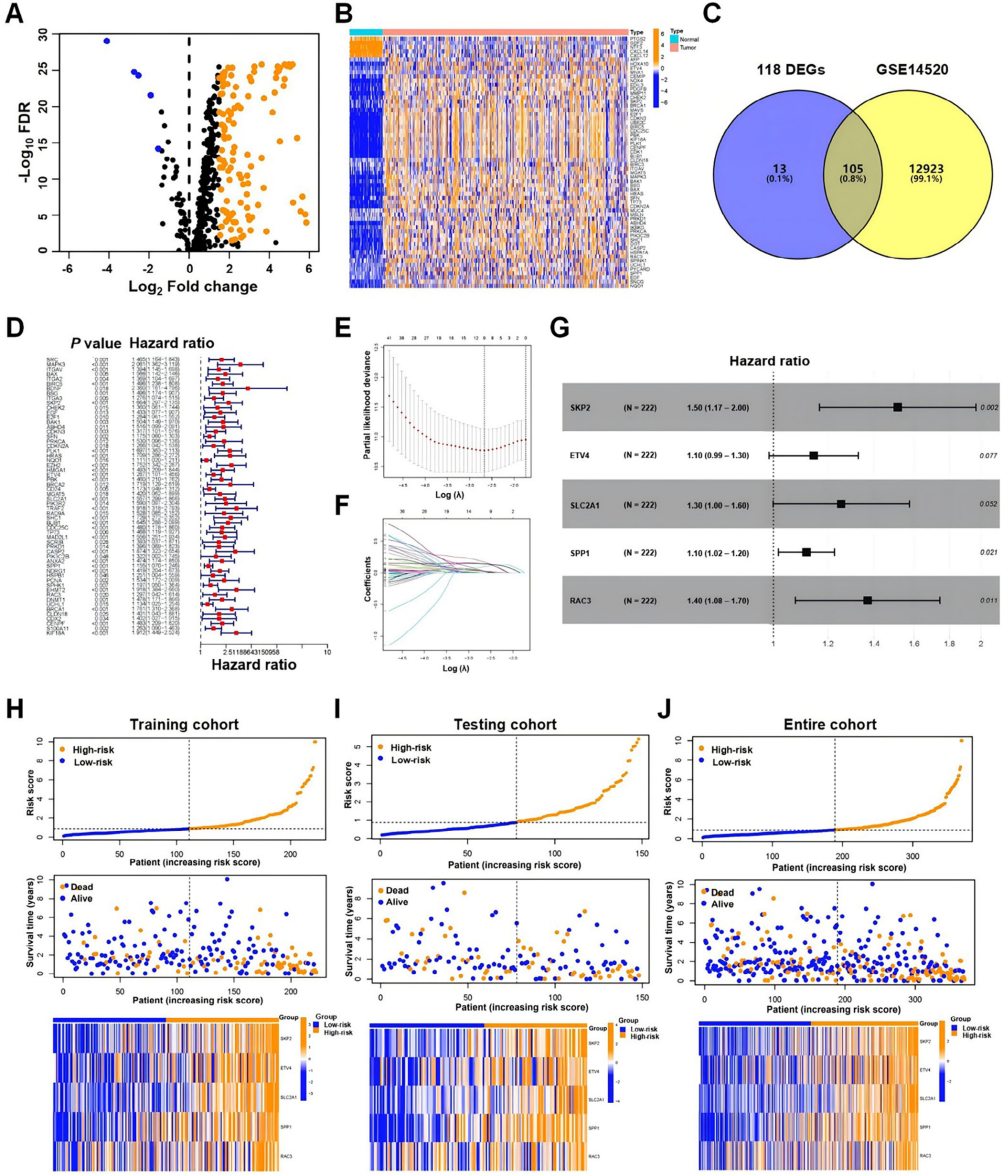
Construction of an ARG signature for patients with HCC. (A) Volcano plots of the differential expression of anoikis‐related genes in HCC and adjacent tissues (blue: significant difference in low expression; orange: significant difference in high expression). (B) Heatmap of the expression of 60 representative DEGs. (C) Venn diagram of the intersection of the GSE14520 and 118 differentially expressed ARGs. (D) Forest plot of 58 ARGs associated with the OS of HCC patients based on univariate Cox analysis. (E) Analysis of the partial likelihood deviation of the LASSO regression. (F) Analysis of the LASSO coefficient. (G) Five ARGs for prognostic model construction based on multivariate Cox analysis. Risk scores and expression levels of five ARGs in the high‐ and low‐risk groups in the training cohort (H), testing cohort (I), and entire cohort (J).

An analysis was conducted on the mRNA expression profiles of 480 ARGs, sourced from GeneCards within the TCGA_LIHC dataset and identified 118 genes that showed differential expression between HCC tissues and their adjacent normal counterparts. Among these genes, 113 genes displayed increased expression levels, whereas five genes showed decreased expression in tumor tissues (Figure 1A), and the heatmap showed representative differentially expressed genes (DEGs) (Figure 1B). By intersecting the HCC dataset GSE14520 with 118 DEGs, we found that 105 genes were common to the two datasets (Figure 1C), which were selected for construction of risk model. In order to investigate the clinical significance of the 105 genes, first, we randomly divided 370 HCC cases from TCGA_LIHC into two groups: a training cohort comprised of 222 cases and a testing cohort made up of 148 cases, which showed balanced clinical features (Table S1); subsequently, we applied univariate Cox regression analysis to the training cohort to determine the prognostic ARGs and 58 out of 105 ARGs were significantly associated with OS (Figure 1D).

### 2.2 Unsupervised Clustering for Patients With HCC Based on ARGs

We used 58 ARGs to conduct a subtype classification of patients with HCC. By incrementally increasing the clustering variable (*k*) from 2 to 9, our observations indicated that the highest degree of similarity within groups (intragroup correlation) and the lowest level of similarity between groups (intergroup correlation) were achieved at *k* = 2. This optimal point suggested that the 370 patients with HCC could be effectively segregated into two distinct clusters (C1 and C2) based on the expression profiles of the 58 ARGs (Figure S1A). The cluster C1 with low ARGs expression had longer OS than that of the cluster C2 with high ARGs expression (Figure S1B) (*p* < 0.001). The heatmap illustrated the transcript expression profiles of 58 ARGs within clusters C1 and C2 (Figure S1C). Further analysis was carried out on the DEGs between the clusters C1 and C2 and 2430 DEGs were identified (Figure S1D). The 2430 DEGs were predominantly involved in pathways including cytokine–cytokine receptor interaction, cell cycle, and extracellular matrix–receptor interaction (Figure S1E). GSEA analyses revealed that pathways including cytokine‐cytokine receptor interaction and ECM receptor interaction were largely overrepresented in the cluster C2, whereas the fatty acid metabolism pathway was predominantly enriched in the cluster C1 (Figure S1F). These findings indicate that based on the expression of ARGs, patients with HCC in different subtype groups display distinct molecular patterns in HCC progression.

### 2.3 Establishment and Verification of ARGs‐Based Risk Model

To explore the ARGs signature for HCC, we incorporated 58 ARGs into LASSO Cox regression and multivariate Cox regression in a stepwise approach to establish a risk model for forecasting survival outcomes in HCC. Five genes (S‐phase kinase associated protein 2 [SKP2], ETS variant transcription factor 4 [ETV4], solute carrier family 2 member 1 [SLC2A1], secreted phosphoprotein 1 [SPP1], and RAC3) of the 58 ARGs strongly correlated with OS were ultimately screened through cross‐validation (Figure 1E, F), which were utilized to construct the risk model in the following manner: risk score = (0.415 × SKP2 expression) + (0.134 × ETV4 expression) + (0.226 × SLC2A1 expression) + (0.110 × SPP1 expression) + (0.314 × RAC3 expression) (Figure 1G). In the TCGA_LIHC training cohort, the median risk score was calculated at 0.911; patients with scores below this threshold were divided into the low‐risk group, while those with scores of 0.911 or higher were categorized into the high‐risk group. The high‐risk cohort exhibited greater expression levels of the five ARGs, and a trend was observed where the OS declined as the risk score escalated (Figure 1H). When the risk model was applied to the testing and the entire cohorts (TCGA_LIHC 370 cases), it produced outcomes consistent with the training cohort, thus validating its reliability (Figures 1I, J).

**FIGURE 2:**
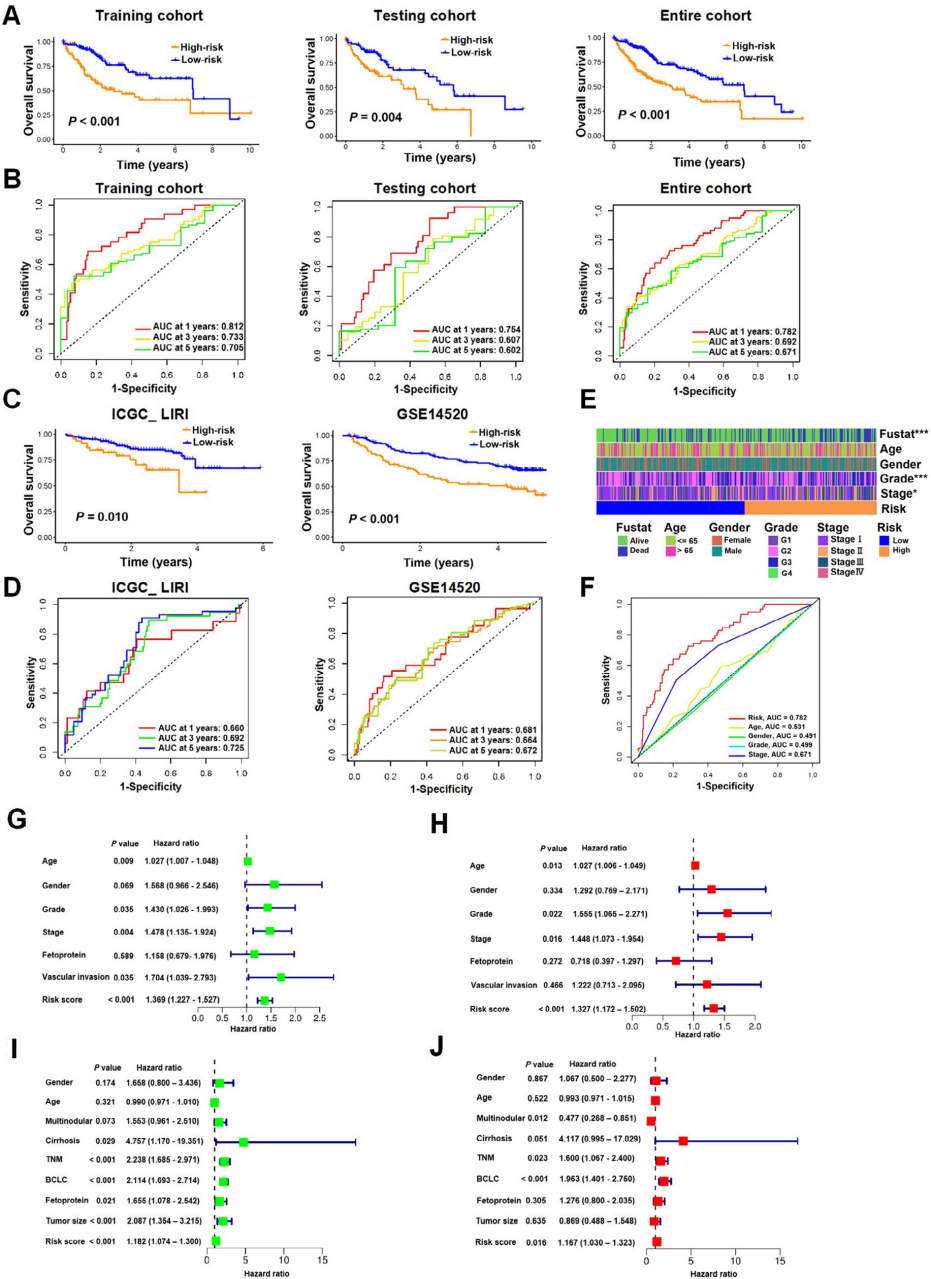
ARG signature in prediction of HCC diagnosis and prognosis. Kaplan–Meier survival (A) and ROC analyses (B) in the training cohort, testing cohort, and entire cohort in the TCGA_LIHC. Kaplan–Meier survival (C) and ROC analyses (D) in the external datasets ICGC_LIRI and GSE14520. (E) Relationships between clinical characteristics and the ARG scores. Tumor grade is categorized as G1: well differentiated, G2: moderately differentiated, G3: poorly differentiated, and G4: undifferentiated. Stage refers to the criteria from the American Joint Committee on Cancer (AJCC). (F) ROC analysis of 1‐year OS stratified by multiple clinical characteristics of HCC patients. Univariate (G) and multivariate (H) Cox analyses of ARGs and prognosis in TCGA_LIHC. Univariate (I) and multivariate (J) Cox analyses of ARGs and patient prognosis in GSE14520. AFP, fetoprotein; BCLC, Barcelona Clinic Liver Cancer. *p < 0.05, ***p < 0.001.

Individuals in the high‐risk group demonstrated notably inferior OS compared to those in the low‐risk group across all three cohorts (Figure 2A). The receiver operating characteristic (ROC) analysis conducted on the training cohort revealed the robust predictive power of the risk model, with area under the curve (AUC) values of 0.812, 0.733, and 0.705 for predicting 1‐, 3‐ and 5‐year survivals, respectively. Correspondingly, in the testing cohort, the AUC values stood at 0.754, 0.607, and 0.602 for the same survival periods, and in the entire cohort, they were 0.782, 0.692, and 0.671, respectively (Figure 2B). To further substantiate the model's diagnostic prediction accuracy, we employed the external datasets ICGC_LIRI and GSE14520 for additional validation, yielding results that were in alignment with those from the initial training cohort (Figures 2C, D).

### 2.4 ARGs Signature as an Independent Predictor of Prognosis for HCC Patients

We next investigated the correlation between the ARGs signature and clinical characteristics in patients with HCC. Our findings revealed significant disparities in tumor stage, tumor grade, and survival between the high‐risk and low‐risk groups (Figure 2E). Moreover, ROC analysis substantiated that the risk model displayed superior predictive power with an AUC of 0.782, outperforming other clinical attributes including age, gender, grade, and stage in the TCGA_LIHC cohort (Figure 2F).

To ascertain whether the ARGs signature's risk score could serve as an independent prognostic biomarker for HCC patients, we conducted univariate and multivariate Cox regression analyses on HCC cohorts, considering variables such as the risk score and additional clinical features. For the TCGA_LIHC cohort, the univariate analysis indicated that age, tumor grade, tumor stage, vascular invasion, or the risk score was associated with OS (Figure 2G), whereas multivariate analysis confirmed that age, tumor grade, tumor stage, or the risk score independently predicted prognosis in HCC patients (Figure 2H). For the GSE14520 cohort, both univariate and multivariate analyses revealed significant correlations between TNM stage, Barcelona Clinic Liver Cancer (BCLC) stage, or the risk score and OS (Figure 2I, J). These findings collectively suggest that the risk score derived from the ARG signature holds potential as an independent prognostic factor for HCC patients.

### 2.5 Nomogram Construction and Evaluation of Immune Cell Infiltration and Chemotherapy Sensitivity

To enhance the practice of the risk model in clinic, a predictive nomogram was constructed for estimating 1‐, 3‐, and 5‐year OS in the TCGA_LIHC based on clinical features, encompassing gender, tumor grade, age, tumor stage, and risk score of ARGs signature (Figure S2A). ROC analysis showed good accuracy of nomogram with AUC values of 0.752, 0.731, and 0.742 (Figure S2B), respectively, and calibration curves confirmed the nomogram's high accuracy (Figure S2C). These results show that the nomogram can be applied to aid clinical decision‐making and to forecast the OS of HCC patients.

The disparities in the survival of HCC patients could be attributed to the sophistication nature of the tumor immune microenvironment [18]. Our analysis of immune cell infiltration and immune‐related pathways among individuals categorized into high‐ and low‐risk groups revealed a notable increase in dendritic cells, macrophages, Th2 cells, and regulatory T cells within the TCGA_LIHC cohort, particularly in those identified as being at high risk (*p* < 0.05) (Figure S2D), and the immune cell subsets of B cells, mast cells, and NK cells were significantly downregulated (*p* < 0.05) (Figure S2D). In the realm of immune‐related pathways, there was a pronounced increase in the expression of checkpoint and major histocompatibility complex class I molecules in the high‐risk group (*p* < 0.05), whereas the type I and type II interferon responses exhibited significant decrease (*p* < 0.05) (Figure S2E). Upon further scrutiny of the variations in immune subtypes between the high‐ and low‐risk groups, it was observed that the distribution of lymphocyte‐depleted samples was fairly balanced across both groups. However, a higher frequency of inflammatory was noted in the low‐risk group, contrasted with predominances of wound healing and interferon‐γ dominant samples in the high‐risk group (Figure S2F).

Additionally, notable variations were observed in the response to chemotherapeutic agents between the two groups. The IC50 values for docetaxel and gefitinib were significantly higher in the high‐risk group, implying a greater resistance to these medications. Conversely, the IC50 values for gemcitabine and imatinib were found to be lower in the high‐risk group compared to the low‐risk group, signifying a more sensitivity to these drugs (Figure S2G). To sum up, the risk scores deduced from the ARGs signatures are capable of predicting the survival for HCC patients and the response to chemotherapy.

### 2.6 Upregulated RAC3 Is Correlated With Tumor Progression and Poor Prognosis in HCC Patients

Subsequently, we revealed that the levels of mRNA and protein for the five ARGs of risk model were markedly higher in HCC tissues than in the adjacent normal tissues (Figure S3A,B). Furthermore, the increases in five ARGs expression were significantly correlated with reduced OS (Figure S3C). Nevertheless, only RAC3 was statistically associated with worse disease‐free survival (*p* = 0.026) (Figure S3D). Hence, we next focused on the role and mechanisms of RAC3 in HCC tumorigenesis and progression.

The RAC family consists of RAC1, RAC2, and RAC3, in which RAC3 was found to be upregulated significantly and distinctly in HCC compared with adjacent normal tissues (*p* < 0.001, 3.3‐fold) (Figure S4A). Additionally, notable upregulations of RAC3 in tumor tissues were observed in the TCGA pan‐cancer, such as BLCA, BRCA, CESC, COAD, ESCA, HNSC, LIHC, LUAD, LUSC, PCPG, PRAD, STAD, and UCEC (Figure S4B). In contrast, RAC3 levels were significantly reduced in tumor tissues of KICH, KIRC, KIRP, and THCA (Figure S4B). In addition, compared with the low RAC3 expression, the high RAC3 expression was linked to poorer outcomes in individuals with bladder cancer, KIRC, sarcoma, and thyroid carcinoma, but longer survival in breast cancer and PDAC (Figure S4C).

**FIGURE 3:**
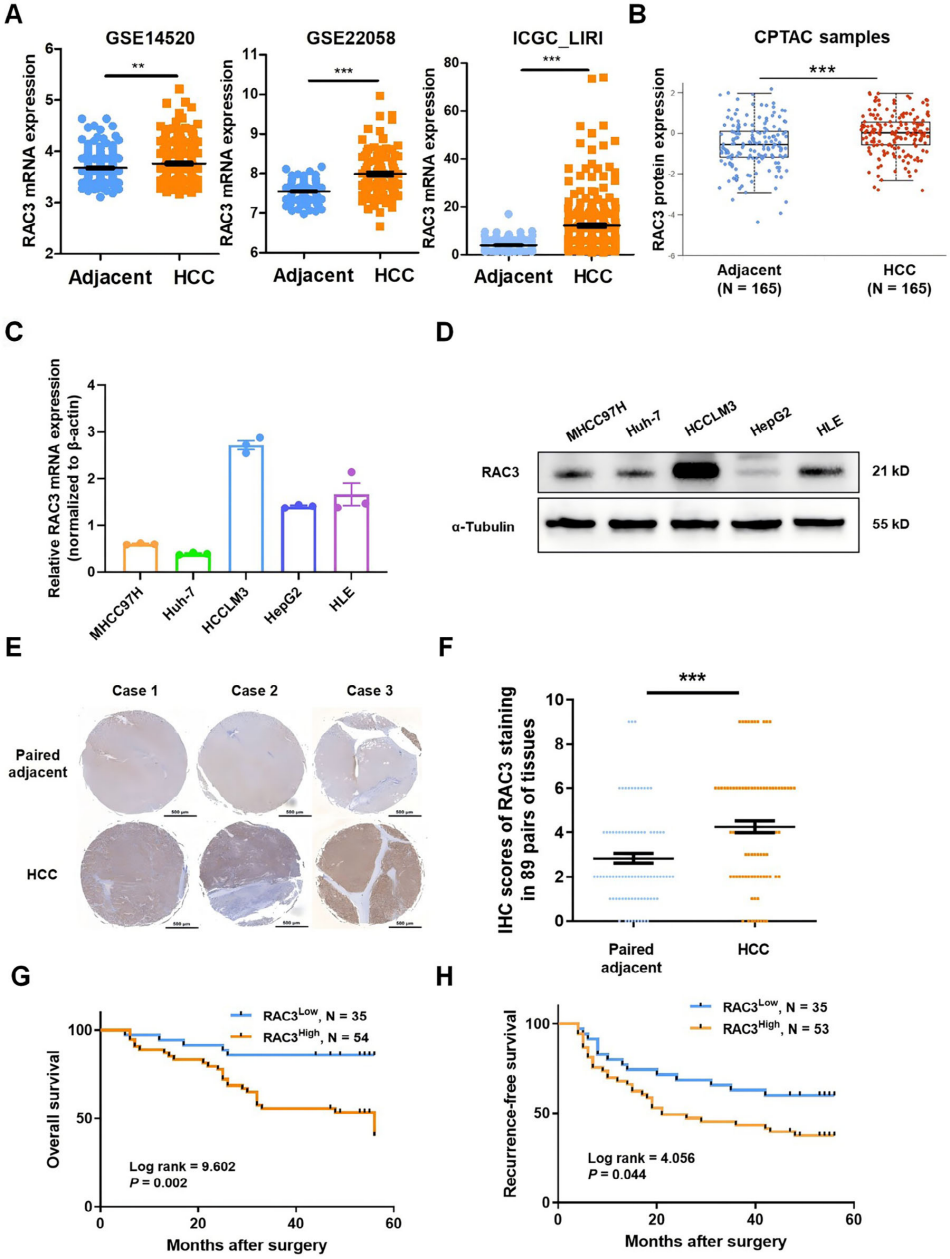
Expression and prognostic significance of RAC3 in HCC tissues. (A) RAC3 mRNA expression of HCC and adjacent normal tissues in three datasets GSE14520 (adjacent: *N* = 220, HCC: *N* = 225), GES22058 (adjacent: *N* = 97, HCC: *N* = 100), and ICGC_LIRI (adjacent: *N* = 202, HCC: *N* = 242). (B) RAC3 protein expression of HCC (*N* = 165) and adjacent normal tissues (*N* = 165) in CTPAC database. (C) qRT‐PCR analysis of RAC3 expression in five HCC cell lines. (D) Western blot analysis of RAC3 expression in five HCC cell lines. (E) Representative IHC images of RAC3 in HCC and paired adjacent normal tissues from tissue microarray. (F) IHC scores associated with RAC3 expression in HCC and paired adjacent normal tissues (*N* = 89). Kaplan–Meier analysis of OS (G) and RFS (H) of HCC patients with high or low RAC3 expression. **p < 0.01, ***p < 0.001. Data represent mean ± SEM collected from three independent experiments.

We conducted analyses of RAC3 expression based on GSE14520, GSE22058, and ICGC_LIRI, which confirmed that RAC3 mRNA was notably higher in HCC tissues compared to the adjacent normal tissues (Figure 3A). Additionally, analysis of the CPTAC database by UALCAN demonstrated that RAC3 protein was notably elevated in HCC tissues compared with adjacent normal tissues (Figure 3B). Kaplan‒Meier plotter analysis revealed that higher expression of RAC3 was associated with poorer prognosis in HCC individuals with stage II and AJCC_T2 (Table S2, Figure S5). These findings strongly indicate that RAC3 can serve as a promising prognostic biomarker for HCC patients.

Subsequently, qRT‐PCR and western blot analyses were carried out to assess the expression levels of RAC3 across five HCC cell lines (Figure 3C, D). Our findings revealed that RAC3 was prominently expressed in HCCLM3 and HLE cells, whereas its expression was considerably lower in MHCC97H and Huh‐7 cells. Moreover, we performed immunohistochemical staining to detect RAC3 expression in an HCC tissue microarray comprised of 89 matched pairs of HCC tumors and their adjacent normal tissues. Similarly, it was observed that RAC3 expression was markedly higher in HCC tissues as opposed to matched normal tissues (Figure 3E, F). Correlation analysis between RAC3 protein expression and clinicopathological parameters indicated significant links between high RAC3 expression and tumors larger than 3 cm (*p* = 0.049), tumor recurrence (*p* = 0.034), and OS (*p* = 0.001) (Table 1). Univariate analysis further demonstrated that tumor size (*p* = 0.016), tumor grade (*p* = 0.027), tumor capsule integrity (*p* = 0.045), TNM stage (*p* = 0.006), and RAC3 expression (*p* = 0.005) were notably related to OS (Table S3). We further conducted a multivariate Cox regression analysis incorporating variables with significance in the univariate analysis, which confirmed that RAC3 protein expression serves as an independent predictor for OS (*p* = 0.044) (Table S4). Kaplan–Meier survival curves demonstrated that higher RAC3 protein expression correlated with significantly poorer prognosis (*p* = 0.002) (Figure 3G) and increased cumulative recurrence‐free survival (*p* = 0.044) (Figure 3H). Collectively, these findings suggest that elevated RAC3 expression plays a pivotal role in the malignancy and progression of HCC.

**TABLE 1:** Association of the RAC3 expression with patients’ clinicopathological features.

Clinicopathological features	RAC3^low^ (*N* = 35)	RAC3^high^ (*N* = 54)	*p* value
Age (*N*, ≤60/>60 years old)	29/6	39/15	0.248
Gender (*N*, male/female)	30/5	49/5	0.463
Tumor size (*N*, ≥3 cm/<3 cm)	22/13	44/10	**0.049**
Tumor number (*N*, 1/>1)	33/2	45/9	0.136
Tumor grade (*N*, I–II/II/II–III/III)	2/16/11/6	1/24/11/18	0.255
Tumor capsule integrity (*N*, yes/no)	17/18	24/29	0.762
TNM stage (*N*, I, II, III)	26/9/0	36/16/2	0.452
Recurrence (*N*, yes/no)	14/21	34/20	**0.034**
HBsAg (*N*, positive/negative)	26/9	43/10	0.445
AFP (*N*, ≥25 µg/L/<25 µg/L)	19/16	31/22	0.697
OS (*N*, live/dead)	30/5	28/26	**0.001**

*Note*: RAC3^low^—IHC score = 0–3; RAC3^high^—IHC score = 4–9); *p* value is estimated by *χ* ^2^ test.

Bold values represent statistically significant (*p* < 0.05).

### 2.7 RAC3 Promotes the Malignant Phenotypes of HCC Cells

**FIGURE 4:**
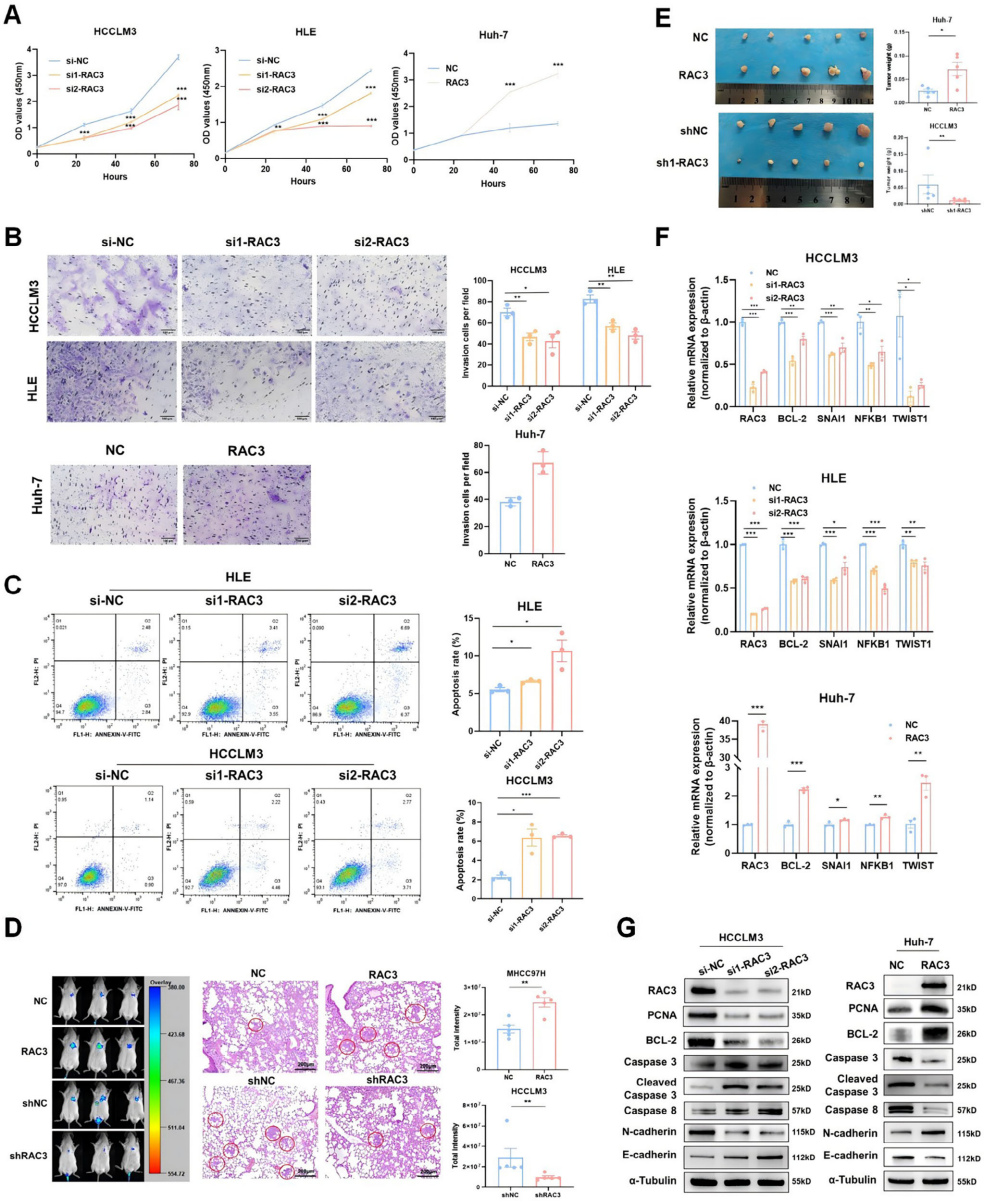
RAC3 promotes the malignant phenotypes of HCC cells. The effect of siRNA knockdown of RAC3 and stable overexpression of RAC3 on the proliferation (A), invasion (B), and apoptosis (C) of HCC cells in vitro. (D) Effects of RAC3 knockdown or overexpression on lung metastasis in NCG mice (*N* = 5). Representative bioluminescence images (left) and hematoxilin‐eosin staining images (middle) of metastasis tumors, and statistics for luciferase intensity in the lung (right). (E) Effects of RAC3 knockdown or overexpression on HCC tumor growth in nude mice (*N* = 5). (F) qRT‐PCR analysis of the expression of anti‐apoptosis markers and (G) western blot analysis of the expression of anti‐apoptosis, proliferation, and EMT markers when RAC3 was knocked down or overexpressed. *p < 0.05, **p < 0.01, ***p < 0.001. Data represent mean ± SEM collected from three independent experiments.

GSEA enrichment based on TCGA_LIHC revealed that RAC3 expression was positively correlated with APOPTOSIS (ES = 0.54, *p* = 0.00), ECM RECEPTOR INTERACTION (ES = 0.68, *p* = 0.00), and CELL ADHESION MOLECULES CAMS (ES = 0.62, *p* = 0.00) (Figure S6A), indicating its potential function in anoikis resistance. Furthermore, to assess the role of RAC3 in HCC, stable overexpression of RAC3 was established in the Huh‐7 cell line, and RAC3 knockdown was performed in the HCCLM3 and HLE cell lines. Knockdown of RAC3 significantly inhibited cell proliferation and invasion in vitro, while overexpression of RAC3 promoted these behaviors (Figure 4A, B). Furthermore, we established stable knockdown of RAC3 in the HCCLM3 cell line (Figure S6B) and also found the inhibition of RAC3 knockdown on the cell proliferation and invasion (Figure S6C,D). The apoptosis assay revealed a significant elevation in apoptosis rate following RAC3 suppression (Figure 4C).

We then constructed a tumor metastasis model that MHCC97H or HCCLM3 stably expressing firefly luciferase with RAC3 stable overexpression or silencing was injected via the tail vein into NCG (NOD/ShiLtJGpt‐Prkdcem26Cd52Il2rgem26Cd22/Gpt) mice. We discovered that luciferase intensity in the lung was significantly increased by RAC3 overexpression while depressed by RAC3 knockdown (Figure 4D). Nude mice were inoculated subcutaneously with Huh‐7 cells featuring stable RAC3 overexpression and HCCLM3 cells with stable RAC3 knockdown. As depicted in Figure 4E, RAC3 overexpression markedly escalated the tumor mass by day 21 post‐inoculation. Conversely, RAC3 knockdown substantially diminished the subcutaneous tumorigenesis by day 14 post‐inoculation. Immunohistochemistry analysis corroborated that high expression of Ki‐67 was detected in RAC3 overexpression tumor tissues, while the opposite effect was observed in the RAC3 knockdown group (Figure S7A,B).

Moreover, qRT‐PCR analysis revealed that knockdown of RAC3 led to decreased expression of anti‐apoptotic markers, including Bcl‐2, SNAI1, NFKB1, and TWIST1, and the opposite effect was observed in RAC3‐overexpressing Huh‐7 cells (Figure 4F). Finally, western blot analysis demonstrated that RAC3 knockdown led to decreased expression of the proliferative marker PCNA, the anti‐apoptotic marker Bcl‐2, and elevated expression of the caspase‐mediated apoptosis while the opposite was observed in RAC3‐overexpressing Huh‐7 cells (Figure 4G). In summary, RAC3 functions as a key accelerant in the initiation and progression of HCC.

### 2.8 RAC3 Activates cAMP/MAPK/Rap1 Signaling by Upregulation of NNMT

**FIGURE 5:**
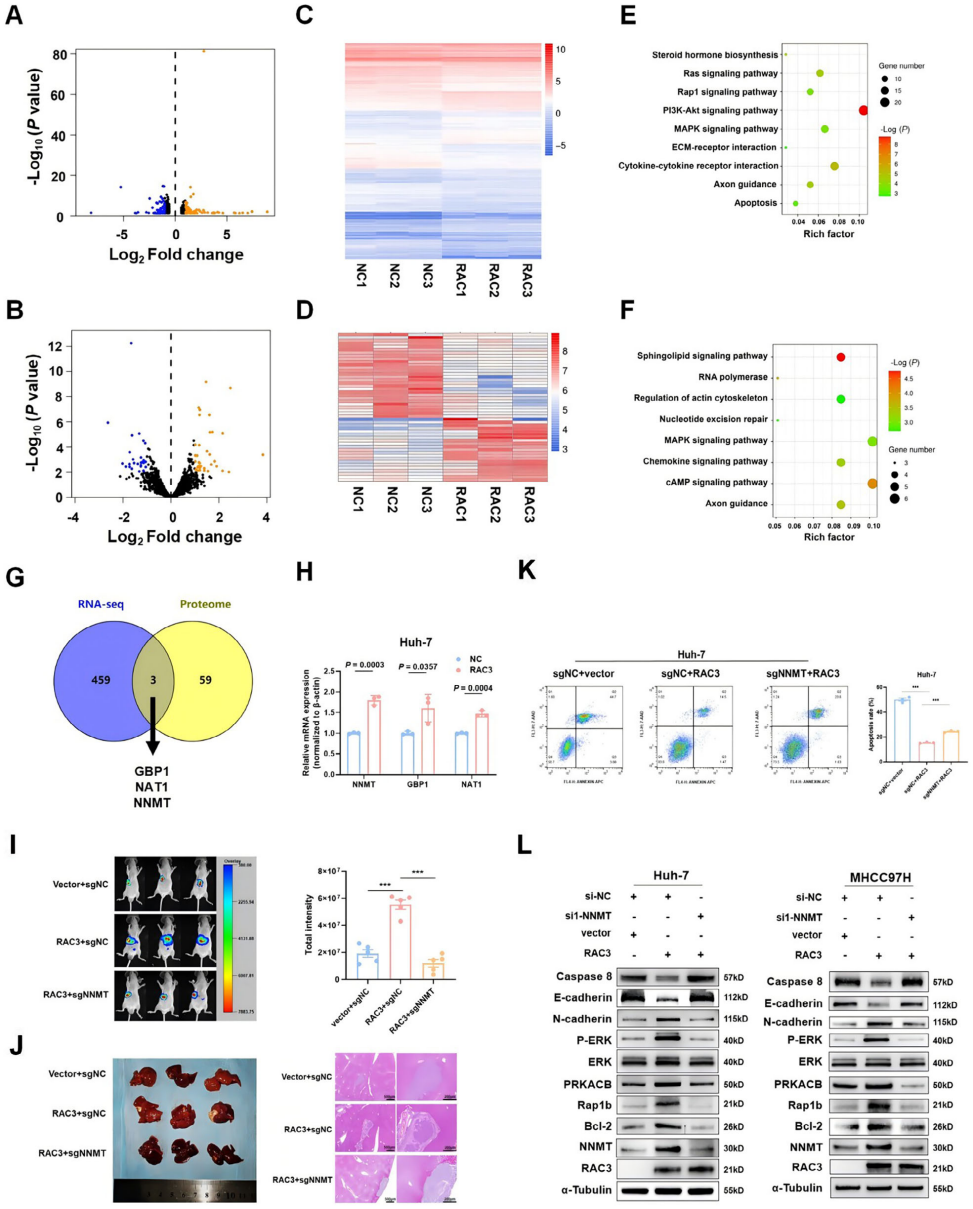
RAC3 activates cAMP/MAPK/Rap1 signaling by upregulation of NNMT. (A and B) Volcano plots showing the DEGs regulated by the overexpression of RAC3 analyzed by RNA‐seq and proteome analysis (blue: significant difference in low expression; orange: significant difference in high expression). (C and D) Heatmaps showing the representative DEGs modulated by the overexpression of RAC3 analyzed by RNA‐seq and proteome analysis. (E and F) KEGG pathway analysis showing the most enriched pathways of DEGs identified by RNA‐seq and proteome analysis. (G) The intersection of 462 and 62 DEGs from the RNA‐seq and proteome data. (H) qRT‐PCR validation of the expression of the three commonly upregulated genes in Huh‐7 cells. (I) Effects of NNMT knockdown on tumor growth in nude mice bearing RAC3 overexpressed MHCC97H cells (*N* = 5). (J) Representative liver tissues (left) and hematoxylin–eosin staining (right). (K) The effect of NNMT knockout on the apoptosis in RAC3 overexpressed HCC cells. (L) Western blot analysis of the expression of MAPK, p‐MAPK, PRKACB, and Rap1b and apoptosis and EMT markers in HCC cells. ***p < 0.001. Data represent mean ± SEM collected from three independent experiments.

To explore the mechanisms by which RAC3 facilitates the HCC progression, we performed RNA sequencing combined with proteome analysis to pinpoint RAC3‐regulated genes. After the overexpression of RAC3, 236 genes and 27 proteins were downregulated, while 226 genes and 35 proteins were upregulated, respectively (Figure 5A, B). Representative DEGs were shown as heatmaps (Figure 5C, D). KEGG analysis based on RNA‐seq data indicated that the Rap1 signaling pathway (*p* = 3.16e‐2), MAPK signaling pathway (*p* = 3.14e‐2), and cytokine‒cytokine receptor interaction (*p* = 5.71e‐3) were significantly enriched (Figure 5E). KEGG analysis based on proteome data revealed that the cAMP signaling pathway (*p* = 1.70e‐2), chemokine signaling pathway (*p* = 3.92e‐2), and MAPK signaling pathway (*p* = 5.02e‐2) were significantly enriched (Figure 5F). Moreover, we intersected 462 DEGs in the RNA‐seq data and 62 DEGs in the proteome and found that three genes, GBP1, NAT1, and NNMT, were commonly upregulated by RAC3 overexpression (Figure 5G). qRT‐PCR was then conducted to monitor the three genes. The outcomes highlighted that NNMT was most significantly upregulated under RAC3 overexpression, given the *p* value and fold change (Figure 5H). Previous studies indicated that NNMT, characterized by elevated expression in HCC, had the capacity to augment the invasion and metastasis of HCC cells, coupled with a correlation to inferior prognosis [19]. Moreover, NNMT was reported to facilitate resistance to ROS‐induced apoptosis in colorectal carcinoma [20]. Thus, we hypothesized that NNMT might act as a key regulator of RAC3‐mediated HCC progression via cAMP/MAPK/Rap1 signaling pathway.

To test the hypothesis, an orthotopic liver HCC model was established by in situ injecting MHCC97H cells in which firefly luciferase and RAC3 were stably overexpressed coupled with NNMT knockout. We found that NNMT knockout reversed the role of RAC3 in promoting HCC growth (Figure 5I, J) and inhibited the expression of Ki67 and Bcl‐2 in RAC3‐overexpression HCC tissues (Figure S8A,B). Additionally, we also performed apoptosis assay to validate that NNMT KO increased apoptosis in RAC3‐overexpression HCC cells in vitro (Figure 5K). Furthermore, we validated the efficiency of three NNMT siRNAs and chose si1‐NNMT to perform NNMT rescue experiments (Figure S8C). We found that PRKACB (key molecule in cAMP signaling), p‐ERK/ERK (key molecules in MAPK signaling), and Rap1b (key molecule in Rap1 signaling) were upregulated through RAC3 overexpression in HCC cells, which could be inhibited by NNMT knockdown (Figure 5L). Moreover, western blot analysis also indicated that the anti‐apoptotic and EMT markers were activated by RAC3 overexpression, which was abolished by NNMT knockdown (Figure 5L). Taken together, these findings imply that RAC3 may serve as a tumor promoter, driving hepatocarcinogenesis and the malignancy of HCC by activating the cAMP/MAPK/Rap1 signaling pathway through NNMT.

### 2.9 RAC3 Interacts with SOX6 Transcriptionally Activating NNMT

**FIGURE 6:**
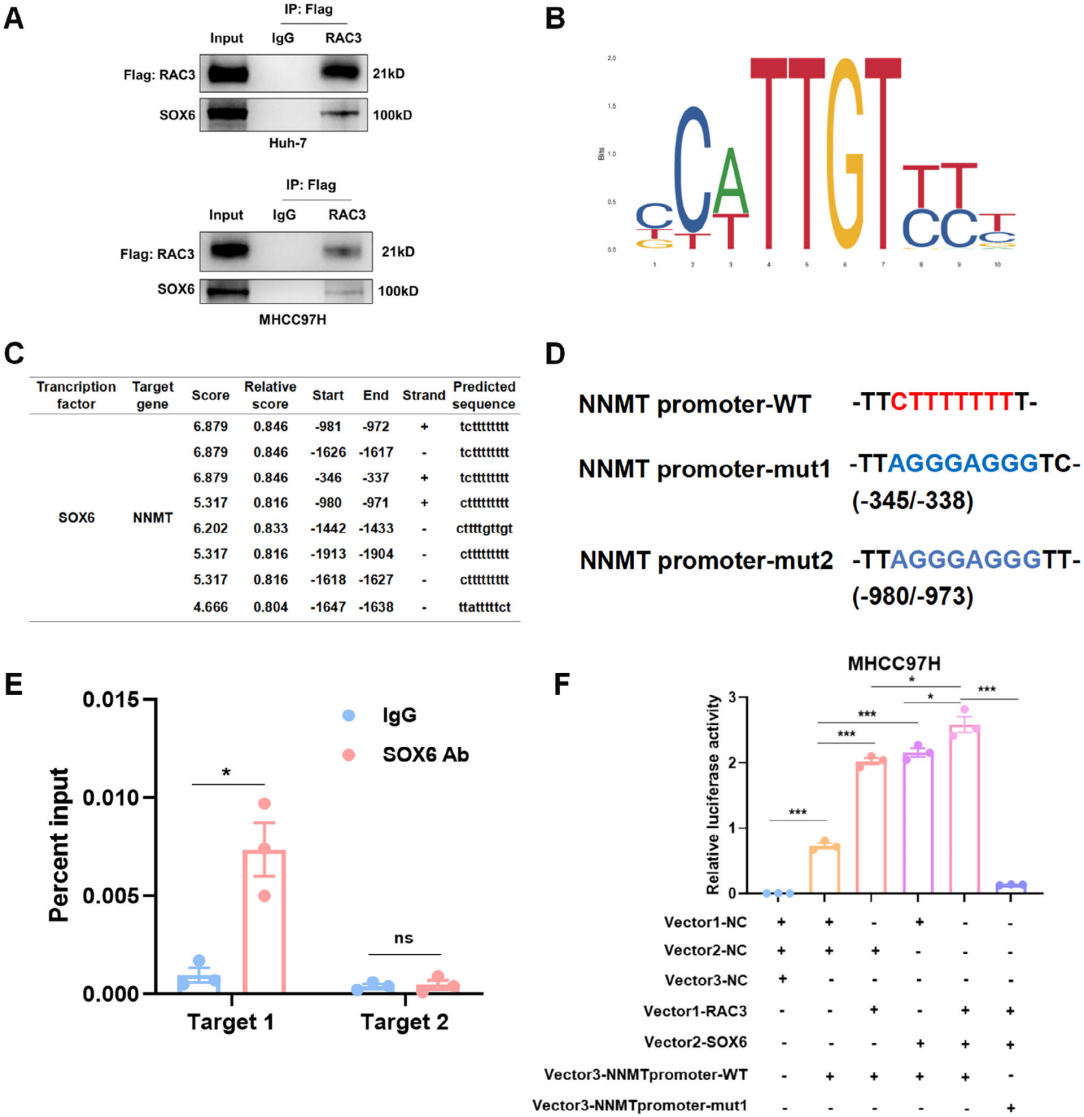
RAC3 interacts with SOX6 to transcriptionally activate NNMT. (A) Co‐immunoprecipitation of SOX6 with anti‐Flag (RAC3) antibody in Huh‐7 and MHCC97H cells. (B) SOX6 core motif binding to NNMT promoter predicted by the Japsar database. (C) DNA sequences binding to SOX6 in the 2000 bp upstream of NNMT (promoter region) predicted by the Jaspar database. (D) The predicted binding sites of SOX6 in the mouse NNMT promoter homologous to human by Jaspar. WT, wild type. (E) ChIP analysis of SOX6 binding at the NNMT promoter in MHCC97H cells. (F) Luciferase activity of the wild type or mutated NNMT promoter reporter after co‐transfection with RAC3 and SOX6 in MHCC97H cells. *p < 0.05, ***p < 0.001, ns, not significant. Data represent mean ± SEM collected from three independent experiments.

It has been reported that RAC3 indirectly activated the transcription of target genes by interacting with transcription factors [17, 21]. Therefore, we hypothesized that RAC3 might act as a transcriptional co‐activator to enhance the transcriptional activity of NNMT and thus promote HCC progression. Consequently, we carried out co‐immunoprecipitation (Co‐IP) combined with mass spectrometry analysis in RAC3‐overexpressed Huh‐7 cells to screen transcription factors interacting with RAC3 (Table S5). Among the RAC3‐interacting proteins, SOX6 was the only annotated transcription factor, which was further predicted to be NNMT‐binding transcriptional factor by Jaspar database. We then verified the interaction between RAC3 and SOX6 in HCC cell lines by Co‐IP (Figure 6A). Due to unavailable of human SOX6 motif in Jaspar database, we predicted the core motif of mouse SOX6 that might bind to the mouse NNMT promoter region (Figure 6B). We found two DNA sequences of the mouse NNMT promoter, homologous to humans, probably binding to SOX6 (Figure 6C, Figure S9). We mutated the two sequences (named mut 1 and mut 2) and designed two pairs of primers (target 1 and target 2) to detect the two sequences using chromatin immunoprecipitation (ChIP)‐qPCR (Figure 6D). The results indicated that SOX6 could directly bind to the NNMT promoter (−345/−338 bp) (Figure 6E). Furthermore, the dual‐luciferase reporter gene assays demonstrated that RAC3 enhanced SOX6‐regulated NNMT transcription, which was significantly inhibited with NNMT promoter mut1 (−345/−338 bp) (Figure 6F). In summary, RAC3 acts as a transcriptional co‐activator of SOX6 to promote NNMT transcription.

### 2.10 Specific Inhibitor EHop‐016 Targeting RAC3 Suppresses HCC Progression

**FIGURE 7:**
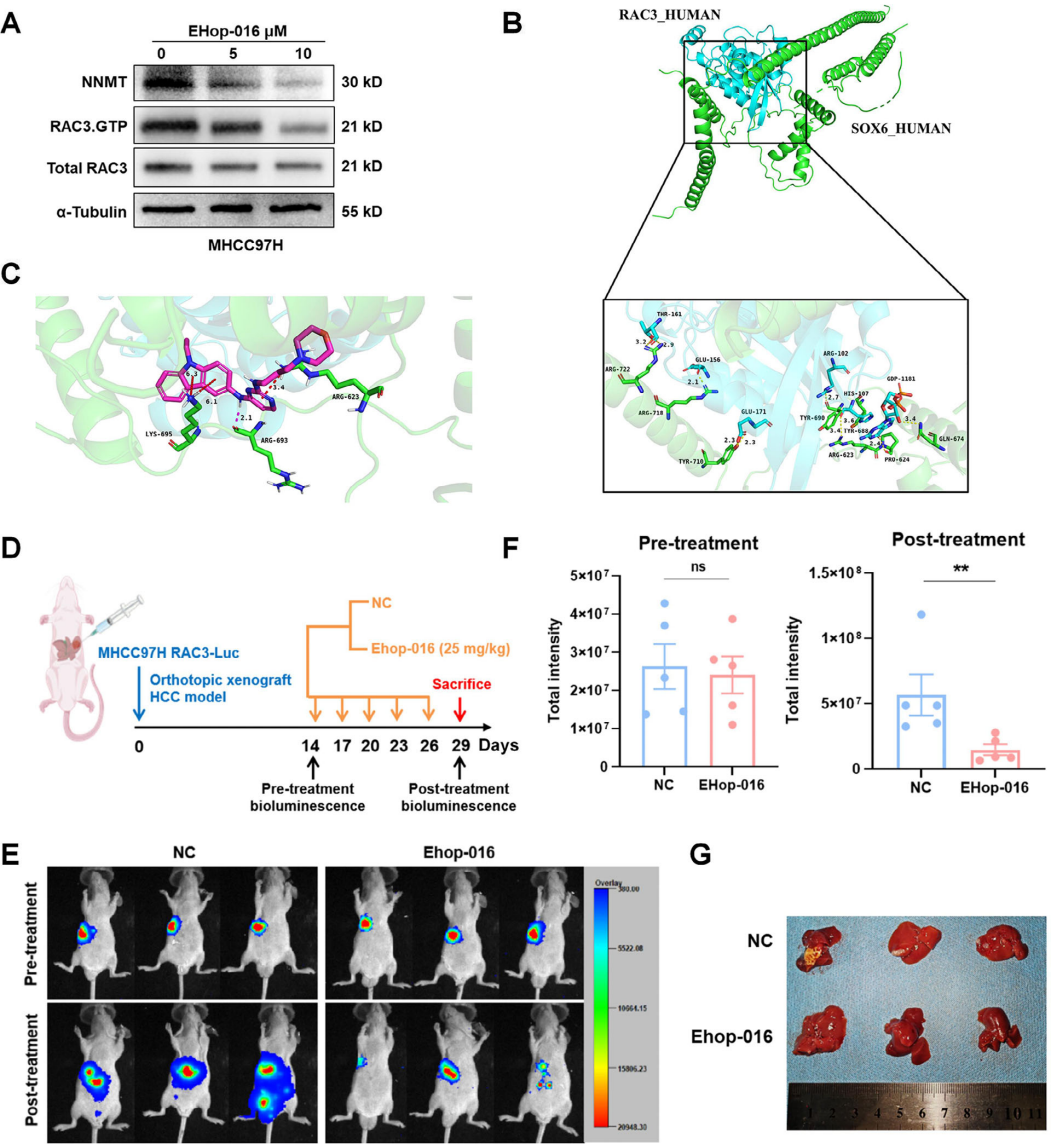
EHop‐016 targeting RAC3 inhibits HCC progression. (A) Western blot analysis of the NNMT expression and RAC3 activity treated with EHop‐016 for 24 h in MHCC97H cells. (B) Molecular docking of RAC3 and SOX6. (blue: RAC3 protein, green: SOX6 protein, yellow dotted line: hydrogen bonding). The numeric value represents the bond length. (C) Molecular docking of EHop‐016 with the interaction interface of RAC3 and SOX6 (blue: RAC3 protein, green: SOX6 protein, pink: EHop‐016, pink dotted line: hydrogen bonding, red dotted line: π‐π interaction). The numeric value represents the bond length. (D) Flow chart of an orthotopic HCC model in nude mice (*N* = 5) injected intraperitoneally with EHop‐016 and saline (NC). (E) Representative bioluminescence images of orthotopic tumors in nude mice before and after treatment with EHop‐016. (F) Statistical diagram of total bioluminescence intensity in orthotopic tumors of nude mice before and after treatment with EHop‐016. (G) Representative tumor tissues after treatment with EHop‐016. **p < 0.01, ns, not significant. (F) Mean ± SEM, Student's *t* test. Data represent mean ± SEM collected from three independent experiments.

We then investigated the intervention efficacy targeting RAC3. Small molecular inhibitor EHop‐016 has been reported to specifically target RAC3 GTPase [22]. Subsequently, MHCC97H cells were treated with EHop‐016 at different concentrations for 24 h. We discovered that EHop‐016 inhibited the enzyme activity of RAC3 and the expression of NNMT in a concentration‐dependent manner (Figure 7A).

Further, we carried out molecular docking based on the three‐dimensional (3D) structures of RAC3, SOX6, and EHop‐016, and found that the hydrogen bond force between RAC3 and SOX6 was about 3 Å, indicating a strong binding (Figure 7B). Moreover, EHop‐016 bound to the interaction interface between RAC3 and SOX6, suggesting that EHop‐016 might disrupt their interaction by inhibiting the activity of RAC3 (Figure 7C). To further evaluate the in vivo pharmacodynamics of EHop‐016, a mouse model with orthotopic implantation of RAC3‐Luc overexpressing MHCC97H cells was intraperitoneally injected with EHop‐016 every 3 days for a total of five times (Figure 7D). We found that the luciferase intensity and the number of luciferase positive lesion in liver was significantly decreased after treatment (Figure 7E–G). Taken together, EHop‐016 is identified as an effective compound targeting RAC3 in HCC therapy.

## 3 Discussion

Anoikis plays a critical role in the initiation and development of HCC [23]. In the present work, a prognostic risk model based on ARGs for patients with HCC was constructed. The risk model segregated the TCGA_LIHC training cohort into two subsets: high risk and low risk. Individuals in the high‐risk category exhibited poorer survival outcomes compared to those in the low‐risk category. Notably, the risk score derived from the model could independently forecast the survival of patients with HCC. Our investigation has yielded several novel insights. First, we successfully constructed a five‐gene risk model, named ARGs signature with high reliability by internal and external cross‐validation, thereby elaborating the importance of ARGs in evaluating the diagnosis and prognosis of HCC patients. Second, the model had high specificity and sensitivity, qualifying it as an independent prognostic indicator to support clinical decision‐making in HCC. Third, RAC3 was uncovered as a novel anoikis‐related oncogene in HCC that promoted malignant phenotypes via NNMT‐mediated activation of cAMP/MAPK/Rap1 signaling. Fourth, this study proposed a strategy to inhibit HCC progression by targeting RAC3.

Several studies have established prognostic models based on ARGs in different types of cancers [24, 25, 26, 27]. In terms of research design, Diao et al. [25] constructed a prognostic ARG signature in LUAD containing of 16 genes. Cai et al. [26] developed a prognostic model for colorectal cancer based on ARGs and immune‐related genes collected from the entire TCGA database. Both studies lacked external dataset cross‐validation. In our study, the risk model was based on 105 DEGs that were common to TCGA_LIHC and GSE14520 in order to make the external validation in GSE14520 go smoothly. Ultimately, the ARGs signature we constructed was refined with five genes, meanwhile internal and external cross‐validation with multiple datasets were performed to ensure model reliability. In terms of application for clinical guidance, our model had high specificity and sensitivity with AUC values of 0.812 for 1‐year survival, greater than other studies. For example, Zhong et al. developed a risk model for HCC based on ARGs and the AUC value of ROC curve was 0.778 for 1‐year survival [27]. Additionally, the study unveiled a substantial correlation between the risk scores of ARGs signature and immune cell infiltration or chemotherapy sensitivity, underscoring the clinical practice of the risk model.

Our risk model included five genes: SKP2, SLC2A1, ETV4, SPP1, and RAC3. SKP2 is an F‐box protein that is a component of the cullin–RING ubiquitin ligase complex [28]. It has been reported that SKP2 facilitates the degradation of the cell cycle regulators P21, P27, and RASSF1A to promote the proliferation of HCC cells [29]. SLC2A1, which encodes the glucose transporter‐1, is highly expressed in HCC. SLC2A1 is reported to facilitate HCC tumorigenesis by increasing intracellular glucose levels, thereby triggering aberrant cellular metabolism [30]. ETV4 is a member of the ETS family [31] that can interact with YAP, resulting in enhanced YAP activation and intranuclear YAP accumulation. In addition, ETV4 promotes HCC growth by enhancing YAP/TEAD4‐mediated transcriptional activation [32]. SPP1 belongs to the SIBLING family and studies have revealed that SPP1 accelerates HCC progression by reinforcing MDA‐9‐induced macrophage migration and angiogenesis [33]. RAC3 encodes a small GTPase of the Rho family that modulates cytoskeletal reorganization [34]. RAC3 enhances proliferation and metastasis in bladder cancer cells by activating the JAK/STAT signaling pathway mediated by PYCR1 [35]. However, the function of RAC3 in HCC tumorigenesis and progression remains unclear.

RAC is associated with multiple cancers, in which context it is universally overexpressed or hyperactivated [36]. RAC1 is regarded as a necroptosis‐related gene that predicts the survival of patients with HCC [37]. RAC2 has been identified as a biomarker with a moderate mutation frequency in HCC [38]. We found RAC3 expression was most obviously increased in HCC among the RAC family. By analysis of the TCGA, ICGC, GEO, and CPTAC databases, we found that RAC3 was both transcriptionally and translationally upregulated significantly in patients with HCC compared to adjacent normal tissues. Moreover, our HCC tissue microarray analysis consistently demonstrated that RAC3 was up‐regulated in HCC and associated with the tumor size, tumor recurrence, and survival. In addition, elevated RAC3 was related to both worse OS and worse recurrence free survival. Several studies have demonstrated that RAC3 facilitates malignant tumor behaviors. For example, RAC3 is hypomethylated, thus inducing cell proliferation and invasion in endometrial cancer [39]. Moreover, it has also been reported that RAC3 inhibits apoptosis in colon cancer to influence chemotherapy sensitivity [40]. In this study, augmenting RAC3 expression fortified the proliferative, invasive, and resistance to apoptosis traits of HCC cells both in vitro and in vivo.

cAMP is a crucial second messenger that has key functions in cell signaling and controls numerous physiological and pathological processes. Adenylyl cyclases catalyze the generation of cAMP, which results in the conversion of ATP to cAMP. By contrast, phosphodiesterases (PDEs) are in charge of cAMP degradation [41]. The homeostasis in cAMP levels is critical for HCC progression. It was reported that high expression of NNMT, which consumed the universal methyl donor, S‐adenosylmethionine, contributed to decreased H3K4me3 modification and m6A modification, leading to reduced mRNA of target gene [42]. Elevated H3K4me3 served as a transcriptional active histone marker for PDE4B [43]. Increased m6A levels at the 3'UTR of PDE1C and PDE4B enhanced their mRNA stability, thus inhibiting cAMP signaling [44]. Hence, we hypothesized that RAC3 interacting with SOX6 transcriptionally regulated NNMT, which consumed universal methyl donor leading to decreased H3K4me3 modification or m6A modification of PDEs mRNA, and thus downregulated PDEs and inhibited cAMP degradation. Consequently, cAMP/MAPK/RAP1 signaling pathway is over‐activated due to accumulated cAMP. PKA, one of major targets of cAMP, promotes HCC invasion and metastasis [45]. PKA inhibitor H89 enhanced ferroptosis susceptibility in lung cancer cells [46]. cAMP binds to two regulatory subunits of the inactive PKA tetramer, leading to the phosphorylation of serine and threonine residues in substrate proteins, which involved in MAPK activation [41]. Considering the multiple physiological functions of cAMP, PKA might be the candidate target of NNMT in cancer therapy.

In essence, our study constructs a prognostic risk model for patients with HCC composed of five ARGs. We prove that the model has high sensitivity and specificity, providing a potent new instrument for the prognosis of HCC patients. More importantly, we identify RAC3 can inhibit apoptosis and promote proliferation and invasion, contributing to HCC progression, which provides novel insight into molecular mechanisms of HCC. Targeting RAC3 by small molecular compound, EHop‐016 shows potential efficiency for HCC therapy. There are still some limitations in our study: first, we will further collect and expand HCC cohorts to validate the reliability of the risk model; second, molecular mechanisms for NNMT‐mediated RAC3 activated pathways are not well established.

## 4 Materials and Methods

### 4.1 Tissue Microarray

A tissue microarray of human HCC samples (Cat. HLivH180Su15) was custom‐designed from Outdo Biotech Co., Ltd. (Shanghai, China). The research was conducted with the approval of the Ethical Committee of Shanghai Outdo Biotech Company, with the ethical approval number YB M‐05‐02. The information of patients, including age and gender, and follow‐up information, such as recurrence free survival and OS, were recorded.

### 4.2 Animal Studies

All animal procedures were authorized by the Animal Ethics Committee of the National Translational Science Center for Molecular Medicine, Fourth Military Medical University (2024‐NTSCMM‐ID001).

### 4.3 Statistical Analysis

The chi‐square test was performed to assess the association between categorical variables and clinical characteristics. Survival analysis in patients with HCC was conducted using the Kaplan–Meier method and log‐rank test. The ARGs signature's prognosis was assessed with univariate and multivariate Cox regression. Pearson's correlation was used for association analysis, and differences between groups were determined by Student's *t*‐test. A *p* value < 0.05 was considered statistically significant.

## Author Contributions

Writing original draft preparation and investigation: D. Wu and Y. Sun. Conceptualization and data curation: Z.K.L. Software and resources: C.H.G, R.Z.S., and M.L. Validation and methodology: R.Y.Z., H.L.W., and C.L. Visualization and formal analysis: Y. Shi, C.Z., Y.T.W., and D. Wei. Project administration: Z.N.C. Funding acquisition, writing‐reviewing, and editing and supervision: H.J.B. All the authors had read and approved the final version of the manuscript.

## Ethics Statement

The study of HCC tissue microarray was conducted with the approval of the Ethical Committee of Shanghai Outdo Biotech Company (YB M‐05‐02). All animal procedures were authorized by the Animal Ethics Committee of the National Translational Science Center for Molecular Medicine, Fourth Military Medical University (2024‐NTSCMM‐ID001).

## Conflicts of Interest

The authors declare no conflicts of interest.

## Supporting information



Supporting Information

## Data Availability

RNA‐sequencing data have been uploaded to GEO (GSE273947). MS proteomics data are available via ProteomeXchange with identifier PXD057450. All codes for bioinformatics analysis was uploaded to Gitee.
