# Supplementary material for: Integrated Analysis of the Anoikis‐Related Signature Identifies Rac Family Small GTPase 3 as a Novel Tumor‐Promoter Gene in Hepatocellular Carcinoma
Source: MedComm (2020). 2025 Mar 22;6(4):e70125. doi: 10.1002/mco2.70125 (PMC11928873; doi:10.1002/mco2.70125)
Supplement: Supplementary file 1 — Supporting Information [file MCO2-6-e70125-s001.pdf]

Integrated analysis of the anoikis-related signature identifies Rac family small GTPase 3 as a novel tumor-promoter gene in hepatocellular carcinoma

Dong Wu[1,2,*], Ze-Kun Liu[1,2,*#], Ying Sun[1,2,*], Chu-Heng Gou[1,2,3], Run-Ze Shang[1,2], Meng Lu[1,2], Ren-Yu Zhang[1,2], Hao-Lin Wei[1,2], Can Li[1,2], Ying Shi[1,2], Cong Zhang[1,2], Yu-Tong Wang[1,2], Ding Wei[1,2], Zhi-Nan Chen[1,2,#], Huijie Bian[1,2,#]

[1]Department of Cell Biology, National Translational Science Center for Molecular Medicine, Fourth Military Medical University, Xi'an, 710032, China.

[2]State Key Laboratory of New Targets Discovery and Drug Development for Major Diseases, Fourth Military Medical University, Xi'an, 710032, China.

[3]Department of Hepatobiliary Surgery, Xijing Hospital, Fourth Military Medical University, Xi'an 710032, China

[*] These authors contributed equally to this work.

[#]Corresponding authors:

E-mail address: hjbian@fmmu.edu.cn (H.J.B), znchen@fmmu.edu.cn (Z.N.C), liuzekun1@fmmu.edu.cn (Z.K.L)

Running title: Anoikis-related gene RAC3 serves as a novel oncogene in HCC

Supplemental Materials and Methods

1 Transcriptome profiles of HCC tissues

The HCC dataset cohorts in our study should meet the following three inclusion criteria: 1. Patients with HCC who have not received radiotherapy, chemotherapy or targeted therapy; 2. The prognosis time, survival status and clinical information of the patients are complete; 3. The sample size of the HCC dataset included in the study exceeded 100 cases, of which the number of patients who died or survived was more than 30. According to the inclusion criteria, we acquired transcriptomic profiles and corresponding clinical details of HCC from the Cancer Genome Atlas (TCGA) repository (encompassing 50 adjacent and 374 HCC tissues) and the International Cancer Genome Consortium (ICGC) repository (encompassing 202 adjacent and 243 HCC tissues). The clinical manifestations such as gender, tumor stage, age, histological grade and survival time were collected from the HCC patient cohort. In addition, we downloaded the HCC microarray expression datasets GSE22058 (encompassing 97 adjacent and 100 HCC tissues) and GSE14520 (encompassing 220 adjacent and 225 HCC tissues) from the Gene Expression Omnibus (GEO) repository.

2 Development of the ARG signature for HCC

The flow chart of machine learning approach applied to the prognostic model analysis was depicted in Figure S10.

2.1 Identification of ARGs and analysis of differentially expressed genes

We conducted a search for genes associated with anoikis utilizing the GeneCards database. The number of ARGs identified, which met the criteria "Category: Protein

Coding" and "Relevance score > 0.4", amounted to 480. The analysis of differential expression was performed using R (version 4.2.1) with package "limma", and the differentially expressed genes were selected in accordance with the specified criteria: false discovery rate (FDR) < 0.05 and $|\log_2 \text{fold change}| \geqslant 1.5$. We used the "pheatmap" package in R for heatmap visualization.

2.2 Random grouping of the cohorts

370 HCC specimens were paired with an identical number of patients, all of whom had comprehensive survival data at their disposal, which were retrieved from the TCGA_LIHC. The entire cohort consisting of 370 patients was allocated into a training cohort (N = 222) and a testing cohort (N = 148) in a randomized manner to obtain the balanced clinical features. Random grouping of data was performed using the package “caret” in R. The approach of random grouping was described in our previous publication [1].

2.3 Establishment and verification of the ARG signature in HCC

In the training cohort, we conducted univariate analysis to determine the prognostic value of ARGs and subsequently constructed an ARG signature for HCC through the integration of LASSO regression and multivariable analyses. The risk score was calculated using the formula: risk score = Σ ($\beta i \times$ Exp (gene i)), where βi represented the coefficient for gene i and Exp (gene i) denoted the expression level of gene i, enabling the calculation of an individualized risk assessment for each HCC patient. The individuals were categorized into distinct prognostic groups of elevated and diminished risk score, with the demarcation threshold established by the median

risk score ascertained from the training cohort. Then, individualized risk scores were determined for each HCC patient within the testing cohort and across the entire cohort, employing a uniform threshold value. The methods of random grouping were described in our previous publication [1]. The approaches of survival analysis and receiver operating characteristic (ROC) analysis were described in our previous publication [1]. The ICGC_LIRI and GSE14520 datasets were employed as additional sets to verify the prognostic value of the ARG model.

2.4 Unsupervised clustering analysis for HCC

We conducted consensus clustering subtype analysis on HCC samples using the R packages "ConsensusClusterPlus" and "limma". The analysis of the associations between the identified clusters and survival outcomes was conducted, leveraging the capabilities of the R packages "survminer" and "survival".

2.5 Gene enrichment analysis

GSEA software was employed to discern the significant pathways associated with ARGs. The specific gene set "c2.cp.kegg. symbols" was chosen further analysis.

2.6 Construction and calibration of the nomogram

A prognostic nomogram was formulated, integrating ARG signature with clinical features (including gender, grade, age, and stage), by utilizing the R packages 'rms' and 'regplot'. This nomogram was devised to predict the 1-, 3-, and 5-year overall survival rates for patients with HCC. Subsequently, ROC and calibration assessment were conducted to ascertain the precision of the established nomogram. The methods used for constructing and calibrating the nomogram were described in our previous

publication [1].

2.7 Immune cell infiltration analysis

We implemented a single-sample GSEA (ssGSEA) method to assess the immune functional enrichment and cell composition in the distinct prognostic subgroups of patient with HCC.

2.8 Prediction of immunotherapeutic response

Drug sensitivity predictions for individual samples were determined by computing the IC50 values through the application of the package pRRophetic in R [2].

3 Cell culture and transfection

The cell line HCCLM3, which is derived from human HCC, was procured from the Cell Bank of the Chinese Academy of Sciences (Shanghai, China). HLE and Huh-7 cells were procured from the Japanese Collection of Research Bioresources (JCRB, Osaka, Japan). The siRNAs for RAC3 and nicotinamide N-methyltransferase (NNMT) were custom-designed and manufactured by GenePharma Co., Ltd. (Shanghai, China) and the specific nucleotide sequences of siRNAs are detailed in Table S6. Lentiviruses carrying RAC3 and sh-RAC3 were custom-made from Genechem Co., Ltd. (Shanghai, China) for constructing stable cell lines. The procedure used for cultures of cell lines and transiently transfected using Lipofectamine 2000 (Invitrogen, CA, USA) were described in our previous publication [3].

4 Immunohistochemistry

The protocol of immunohistochemistry (IHC) was described in our previous publication [3]. The RAC3 primary antibody (Cat. ab129062) was purchased from Abcam (Cambridge, UK). The Ki67 primary antibody (Cat. 27309-1-AP) was obtained from Proteintech (Wuhan, China).

5 Cell proliferation, apoptosis and invasion assays

The protocol used for cell proliferation, apoptosis and invasion were described in our previous publication [3].

6 Quantitative real-time PCR

The protocol used for qRT–PCR was described in our previous publication [3]. The primers applied to qRT–PCR was described in Table S6.

7 Co-IP assay and western blotting

The interaction of RAC3 and SOX6 in Huh-7 and MHCC97H cells was detected by a Co-immunoprecipitation (Co-IP) kit (Cat. 26149) purchased from Pierce (Rockford, IL, USA). The protocol for western blot was described in our previous publication [3]. Antibodies against the following proteins were used: Bcl-2 (Cat. 26593-1-AP), PCNA (Cat. 10205-2-AP), N-cadherin (Cat. 22018-1-AP), E-cadherin (Cat. 20874-1-AP), caspase-3 (Cat. 66470-2-Ig), NNMT (Cat. 15123-1-AP), RAP1B (Cat. 10840-1-AP), PRKACB (Cat. 12232-1-AP) and α-tubulin (11224-1-AP), which were purchased from Proteintech (Wuhan, China) and the antibodies RAC3 (Cat. ab129062) and cleaved caspase-3 (Cat. ab32042) were purchased from Abcam (Cambridge, UK). The antibodies caspase 8 (Cat. 4790S), phospho-MAPK (Erk1/2; Thr202/Tyr204) (Cat. 4377T) and MAPK (Erk1/2) (Cat. 4695T) were obtained from

Cell Signaling Technology (MA, USA).

8 Animal studies

To detect the ability of RAC3 to promote the tumorigenicity of HCC cells, 1 × 10^6 HCCLM3 or Huh-7 cells with stable overexpression or stable silencing of RAC3 were implanted subcutaneously into the flanks of 6-week-old male nude mice, which were sourced from SPF (Beijing) Biotechnology Co., Ltd. (Beijing, China).

To evaluate the ability of RAC3 to accelerate the metastasis of HCC cells *in vivo*, 1 × 10^6 MHCC97H or HCCLM3 stably expressing firefly luciferase with RAC3 stable overexpression or silencing were injected via the tail vein of 6-week-old male NCG (NOD/ShiLtJGpt-$Prkdc^{em26Cd52}Il2rg^{em26Cd22}$/Gpt) mice, purchased from GemPharmatech Co., Ltd (Nanjing, China). Moreover, before sacrifice, the mice were intraperitoneally injected with D-luciferin potassium for in vivo imaging using the Carestream Molecular Imaging System following the manufacturer's instructions.

To assess the capability of RAC3/ NNMT in promoting hepatocarcinogenesis and examine the inhibitory effect of EHop-016 (Cat. HY-12810), purchased from MCE company (NJ, USA), 2 × 10^6 MHCC97H with stable expressing firefly luciferase and stable overexpression RAC3 or NNMT knockout were in situ injected into the liver of 6-week-old male nude mice. Two weeks after inoculation, the mice were then intraperitoneally injected with Ehop-016 (25 mg/kg), once every three days for a total of five times. Besides, the mice were intraperitoneally injected with D-luciferin potassium before and after treatment for in vivo imaging using the Carestream

Molecular Imaging System.

9 RNA sequencing analysis

Three independent RNA samples from stable RAC3-overexpressing Huh-7 cells and their corresponding negative controls were subjected to sequencing by Gene Denovo Biotechnology Co. (Guangzhou, China). Genes with the fold of expression exceeded 1.5 or less than -1.5 with the *P*-value is less than 0.05 were considered as significantly differentially expressed.

10 Mass spectrometry analysis

Proteins were harvested from Huh-7 cells that stably overexpress RAC3 and their negative controls using RIPA lysis buffer (Beyotime, product P0013B), enhanced with a protease inhibitor cocktail (Roche, code 04693159001, EDTA-free) and PMSF (Beyotime, ST505). The protein concentration was standardized to 1 mg/ml. Liquid chromatography tandem mass spectrometry (LC-MS/MS) was employed on an Orbitrap Eclipse mass spectrometer (Thermo Fisher Scientific). The mass spectrometer was set to perform MS/MS fragmentation on the top 20 most abundant ions for each full MS scan. Proteins were identified as differentially expressed when their expression level showed a fold change greater than 2 or less than -2, accompanied by a statistical significance of $P < 0.01$.

11 Chromatin immunoprecipitation (ChIP) assay

A ChIP Kit (Cat. ab500, Abcam) was utilized to conduct the ChIP assay. In brief, the samples were fixed and then fragmented by ultrasound. The DNA fragments with

a length of 200 - 1000 bp were incubated together with beads and either 5 μg of IgG or SOX6 antibodies. Following the DNA purification of the samples, qRT - PCR reactions produced products from the promoter region of the SOX6 gene. Primers based on the predicted binding sites were employed: Target 1: F: 5’-GAGGGTCTCGGGATGTTTGG-3’; R:5’-CATGTTTAAATTTAAACCATTC-3’ and Target 2: F: 5’-CCCTACTCTGTCTCAAAGTG-3’; R: 5’-TGGTGGTAGGCACCTGCAGT-3’.

12 Dual-luciferase reporter assay

The Dual-Luciferase Reporter Assay System (Cat. E1980), purchased from Promega (Shanghai, China) was used to detect the transcription of NNMT regulated by RAC3 and SOX6. 1 μg RAC3/SOX6 expression plasmid, 200 ng NNMT wildtype/ mutant luciferase reporter plasmid, and 10 ng pRL-TK were co-transfected into MHCC97H cells in a 12-well plate. After 24-hour transfection, cells were tested with Promega's Luciferase Assay System (Cat. E5310).

13 Detection of GTP-bound RAC3

p21-activated kinase 1 (PAK1) is the substrate for detecting the enzymatic activity of RAC3 in its GTP-bound form, thus cell lysates were applied to a pull-down assay using GST-PAK1-PBD fusion and western blot was performed for the activated form of GTP-bound RAC3.

14 Molecular docking

The binding site was predicted through downloading the protein structure of RAC3 (with PDB ID: 2C2H) from the Protein Data Bank and that of SOX6

(AlphaFold ID: AF - P35712 - F1) from the Alphafold Protein Structure Database. The HDOCK Server was utilized to acquire the electrostatic and van der Waals interactions between the target and the subject. Python (Version 3.7.7) - Pymol (Version 2.4.0) was employed for docking graphical processing and intermolecular spatial distances (interactions were regarded as significant when the distances were less than 5 Å).

References

[1] Liu ZK, Wu KF, Zhang RY, et al. (2022). Pyroptosis-Related LncRNA Signature Predicts Prognosis and Is Associated With Immune Infiltration in Hepatocellular Carcinoma. Front Oncol. 12:794034, 10.3389/fonc.2022.794034.

[2] Geeleher P, Cox N, and Huang RS (2014). pRRophetic: an R package for prediction of clinical chemotherapeutic response from tumor gene expression levels. PLoS One. 9(9):e107468, 10.1371/journal.pone.0107468.

[3] Liu ZK, Li C, Zhang RY, et al. (2021). EYA2 suppresses the progression of hepatocellular carcinoma via SOCS3-mediated blockade of JAK/STAT signaling. Mol Cancer. 20(1):79, 10.1186/s12943-021-01377-9.

Figure S1

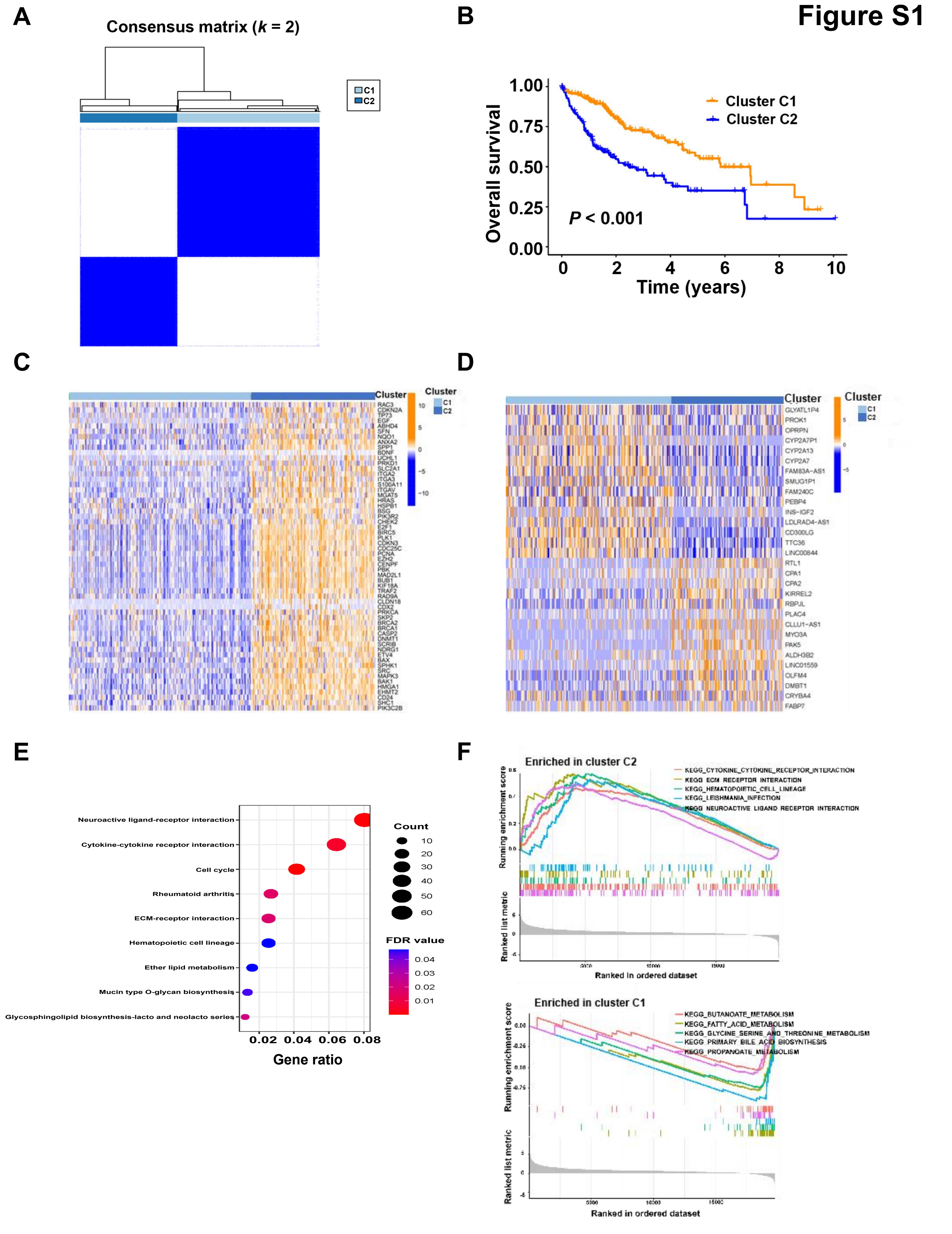

A
Consensus matrix (k = 2)
C1
C2
B
Overall survival
Time (years)
Cluster C1
Cluster C2
P < 0.001
1.00
0.75
0.50
0.25
0.00
0
2
4
6
8
10
C
Cluster
C1
C2
D
Cluster
C1
C2
GLYATL1P4
PROK1
OPRPN
CYP2A7P1
CYP2A13
CYP2A7
FAM83A-AS1
SMUG1P1
FAM240C
PEBP4
INS-IGF2
LDLRAD4-AS1
CD300LG
TTC36
LINC00844
RTL1
CPA1
CPA2
KIRREL2
RBPJL
PLAC4
CLLU1-AS1
MYO3A
PAK5
ALDH3B2
LINC01559
OLFM4
DMBT1
CRYBA4
FABP7
E
Neuroactive ligand-receptor interaction
Cytokine-cytokine receptor interaction
Cell cycle
Rheumatoid arthritis
ECM-receptor interaction
Hematopoietic cell lineage
Ether lipid metabolism
Mucin type O-glycan biosynthesis
Glycosphingolipid biosynthesis-lacto and neolacto series
Count
10
20
30
40
50
60
FDR value
0.04
0.03
0.02
0.01
0.02
0.04
0.06
0.08
Gene ratio
F
Enriched in cluster C2
KEGG_CYTOKINE_CYTOKINE_RECEPTOR_INTERACTION
KEGG_ECM_RECEPTOR_INTERACTION
KEGG_HEMATOPOIETIC_CELL_LINEAGE
KEGG_LEISHMANIA_INFECTION
KEGG_NEUROACTIVE_LIGAND_RECEPTOR_INTERACTION
Running enrichment score
Ranked list metric
Ranked in ordered dataset
Enriched in cluster C1
KEGG_BUTANOATE_METABOLISM
KEGG_FATTY_ACID_METABOLISM
KEGG_GLYCINE_SERINE_AND_THREONINE_METABOLISM
KEGG_PRIMARY_BILE_ACID_BIOSYNTHESIS
KEGG_PROPANOATE_METABOLISM
Running enrichment score
Ranked list metric
Ranked in ordered dataset

Figure S2

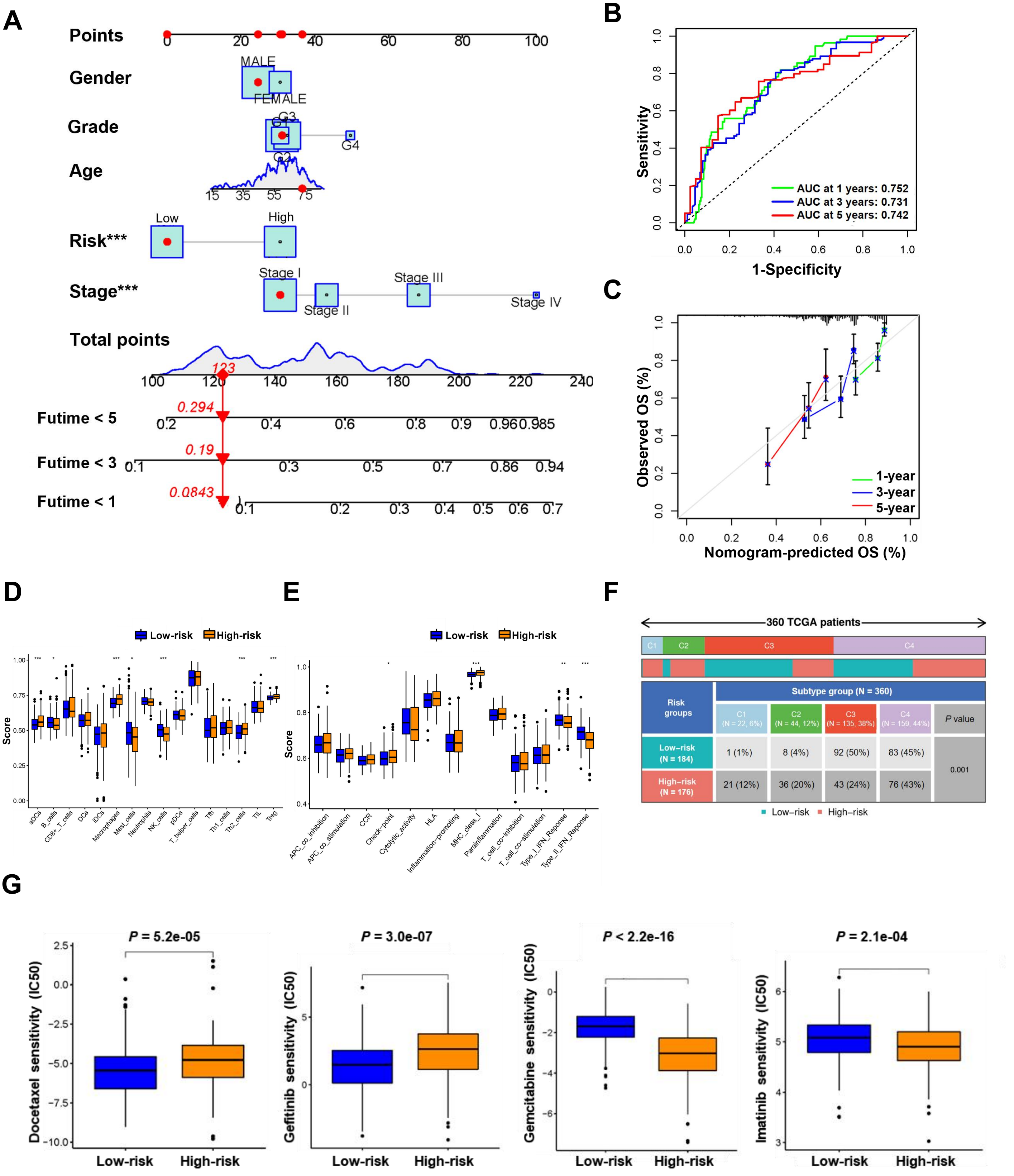
A
Points
Gender
MALE
FEMALE
Grade
G1
G2
G3
G4
Age
Risk***
Low
High
Stage***
Stage I
Stage II
Stage III
Stage IV
Total points
123
Futime < 5
0.294
Futime < 3
0.19
Futime < 1
0.0843
B
Sensitivity
1-Specificity
AUC at 1 years: 0.752
AUC at 3 years: 0.731
AUC at 5 years: 0.742
C
Observed OS (%)
Nomogram-predicted OS (%)
1-year
3-year
5-year
D
Low-risk
High-risk
Score
aDCs
B_cells
CD8+_T_cells
DCs
iDCs
Macrophages
Mast_cells
Neutrophils
NK_cells
pDCs
T_helper_cells
Tfh
Th1_cells
Th2_cells
TIL
Treg
E
Low-risk
High-risk
Score
APC_co_inhibition
APC_co_stimulation
CCR
Check-point
Cytolytic_activity
HLA
Inflammation-promoting
MHC_class_I
Parainflammation
T_cell_co-inhibition
T_cell_co-stimulation
Type_I_IFN_Reponse
Type_II_IFN_Reponse
F
360 TCGA patients
C1
C2
C3
C4
Risk groups
Subtype group (N = 360)
C1 (N = 22, 6%)
C2 (N = 44, 12%)
C3 (N = 135, 38%)
C4 (N = 159, 44%)
P value
Low-risk (N = 184)
1 (1%)
8 (4%)
92 (50%)
83 (45%)
0.001
High-risk (N = 176)
21 (12%)
36 (20%)
43 (24%)
76 (43%)
Low-risk
High-risk
G
P = 5.2e-05
Docetaxel sensitivity (IC50)
P = 3.0e-07
Gefitinib sensitivity (IC50)
P < 2.2e-16
Gemcitabine sensitivity (IC50)
P = 2.1e-04
Imatinib sensitivity (IC50)
Low-risk
High-risk

Figure S3

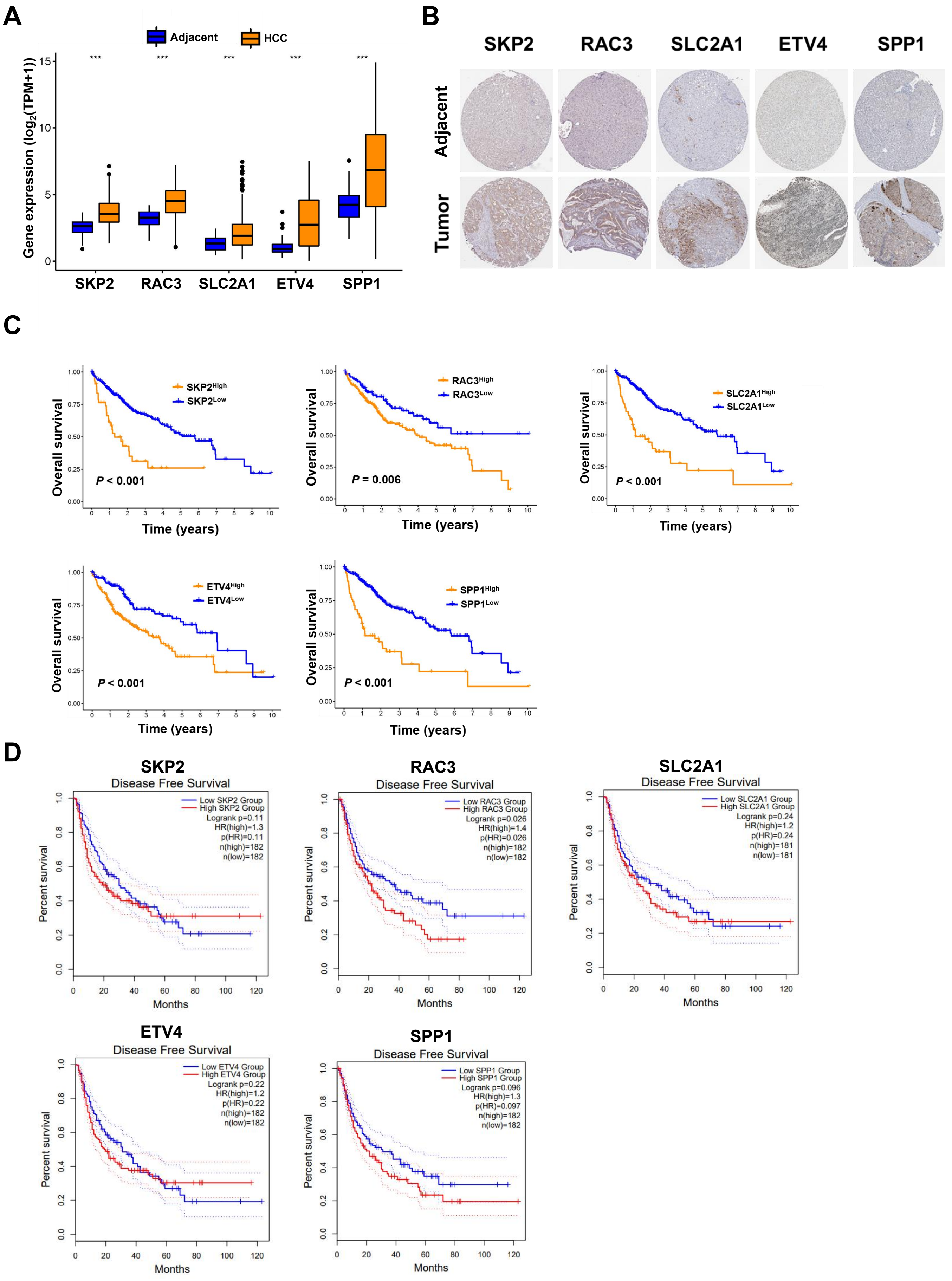

A
Adjacent
HCC
Gene expression (log2(TPM+1))
***
SKP2
RAC3
SLC2A1
ETV4
SPP1
B
SKP2
RAC3
SLC2A1
ETV4
SPP1
Adjacent
Tumor
C
SKP2High
SKP2Low
P < 0.001
RAC3High
RAC3Low
P = 0.006
SLC2A1High
SLC2A1Low
P < 0.001
ETV4High
ETV4Low
P < 0.001
SPP1High
SPP1Low
P < 0.001
Overall survival
Time (years)
D
SKP2
Disease Free Survival
Low SKP2 Group
High SKP2 Group
Logrank p=0.11
HR(high)=1.3
p(HR)=0.11
n(high)=182
n(low)=182
RAC3
Disease Free Survival
Low RAC3 Group
High RAC3 Group
Logrank p=0.026
HR(high)=1.4
p(HR)=0.026
n(high)=182
n(low)=182
SLC2A1
Disease Free Survival
Low SLC2A1 Group
High SLC2A1 Group
Logrank p=0.24
HR(high)=1.2
p(HR)=0.24
n(high)=181
n(low)=181
ETV4
Disease Free Survival
Low ETV4 Group
High ETV4 Group
Logrank p=0.22
HR(high)=1.2
p(HR)=0.22
n(high)=182
n(low)=182
SPP1
Disease Free Survival
Low SPP1 Group
High SPP1 Group
Logrank p=0.096
HR(high)=1.3
p(HR)=0.097
n(high)=182
n(low)=182
Percent survival
Months

Figure S4

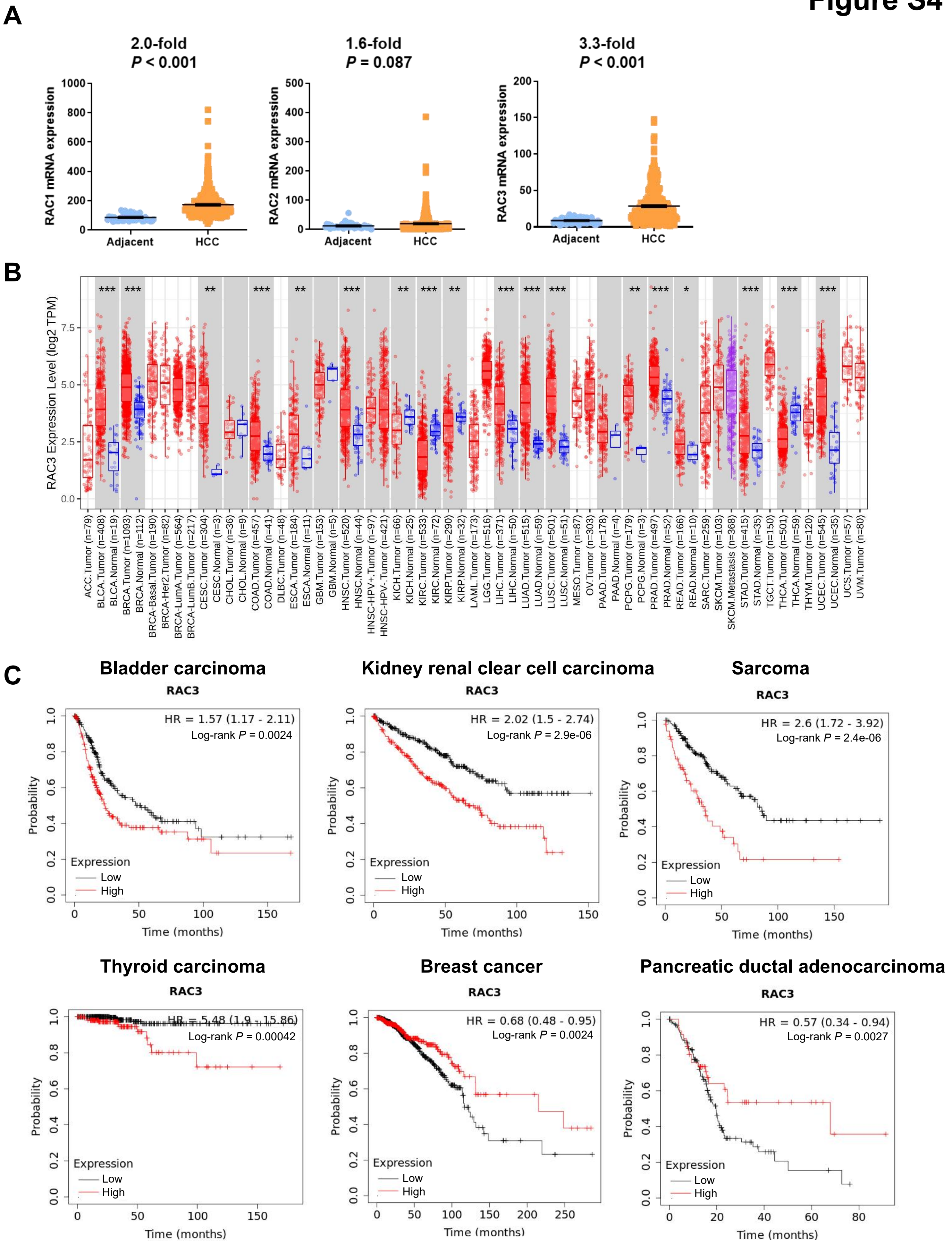

A
2.0-fold
P < 0.001
RAC1 mRNA expression
Adjacent
HCC
1.6-fold
P = 0.087
RAC2 mRNA expression
Adjacent
HCC
3.3-fold
P < 0.001
RAC3 mRNA expression
Adjacent
HCC
B
RAC3 Expression Level (log2 TPM)
ACC.Tumor (n=79)
BLCA.Tumor (n=408)
BLCA.Normal (n=19)
BRCA.Tumor (n=1093)
BRCA.Normal (n=112)
BRCA-Basal.Tumor (n=190)
BRCA-Her2.Tumor (n=82)
BRCA-LumA.Tumor (n=564)
BRCA-LumB.Tumor (n=217)
CESC.Tumor (n=304)
CESC.Normal (n=3)
CHOL.Tumor (n=36)
CHOL.Normal (n=9)
COAD.Tumor (n=457)
COAD.Normal (n=41)
DLBC.Tumor (n=48)
ESCA.Tumor (n=184)
ESCA.Normal (n=11)
GBM.Tumor (n=153)
GBM.Normal (n=5)
HNSC.Tumor (n=520)
HNSC.Normal (n=44)
HNSC-HPV+.Tumor (n=97)
HNSC-HPV-.Tumor (n=421)
KICH.Tumor (n=66)
KICH.Normal (n=25)
KIRC.Tumor (n=533)
KIRC.Normal (n=72)
KIRP.Tumor (n=290)
KIRP.Normal (n=32)
LAML.Tumor (n=173)
LGG.Tumor (n=516)
LIHC.Tumor (n=371)
LIHC.Normal (n=50)
LUAD.Tumor (n=515)
LUAD.Normal (n=59)
LUSC.Tumor (n=501)
LUSC.Normal (n=51)
MESO.Tumor (n=87)
OV.Tumor (n=303)
PAAD.Tumor (n=178)
PAAD.Normal (n=4)
PCPG.Tumor (n=179)
PCPG.Normal (n=3)
PRAD.Tumor (n=497)
PRAD.Normal (n=52)
READ.Tumor (n=166)
READ.Normal (n=10)
SARC.Tumor (n=259)
SKCM.Tumor (n=103)
SKCM.Metastasis (n=368)
STAD.Tumor (n=415)
STAD.Normal (n=35)
TGCT.Tumor (n=150)
THCA.Tumor (n=501)
THCA.Normal (n=59)
THYM.Tumor (n=120)
UCEC.Tumor (n=545)
UCEC.Normal (n=35)
UCS.Tumor (n=57)
UVM.Tumor (n=80)
C
Bladder carcinoma
RAC3
HR = 1.57 (1.17 - 2.11)
Log-rank P = 0.0024
Kidney renal clear cell carcinoma
RAC3
HR = 2.02 (1.5 - 2.74)
Log-rank P = 2.9e-06
Sarcoma
RAC3
HR = 2.6 (1.72 - 3.92)
Log-rank P = 2.4e-06
Thyroid carcinoma
RAC3
HR = 5.48 (1.9 - 15.86)
Log-rank P = 0.00042
Breast cancer
RAC3
HR = 0.68 (0.48 - 0.95)
Log-rank P = 0.0024
Pancreatic ductal adenocarcinoma
RAC3
HR = 0.57 (0.34 - 0.94)
Log-rank P = 0.0027
Probability
Time (months)
Expression
Low
High

Figure S5

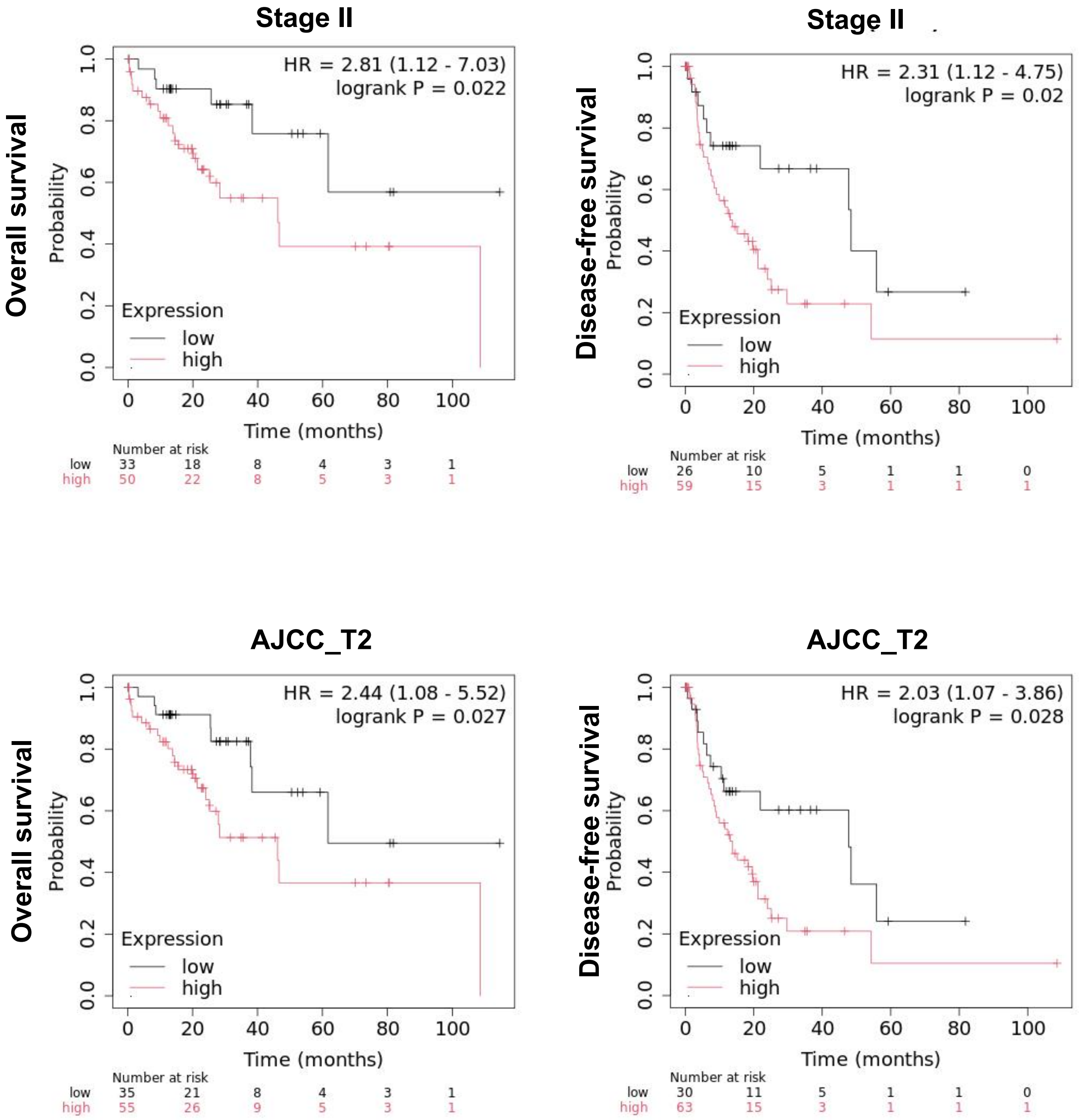
Stage II
Overall survival
Probability
HR = 2.81 (1.12 - 7.03)
logrank P = 0.022
Expression
low
high
Time (months)
Number at risk
low 33 18 8 4 3 1
high 50 22 8 5 3 1
Stage II
Disease-free survival
Probability
HR = 2.31 (1.12 - 4.75)
logrank P = 0.02
Expression
low
high
Time (months)
Number at risk
low 26 10 5 1 1 0
high 59 15 3 1 1 1
AJCC_T2
Overall survival
Probability
HR = 2.44 (1.08 - 5.52)
logrank P = 0.027
Expression
low
high
Time (months)
Number at risk
low 35 21 8 4 3 1
high 55 26 9 5 3 1
AJCC_T2
Disease-free survival
Probability
HR = 2.03 (1.07 - 3.86)
logrank P = 0.028
Expression
low
high
Time (months)
Number at risk
low 30 11 5 1 1 0
high 63 15 3 1 1 1

Figure S6

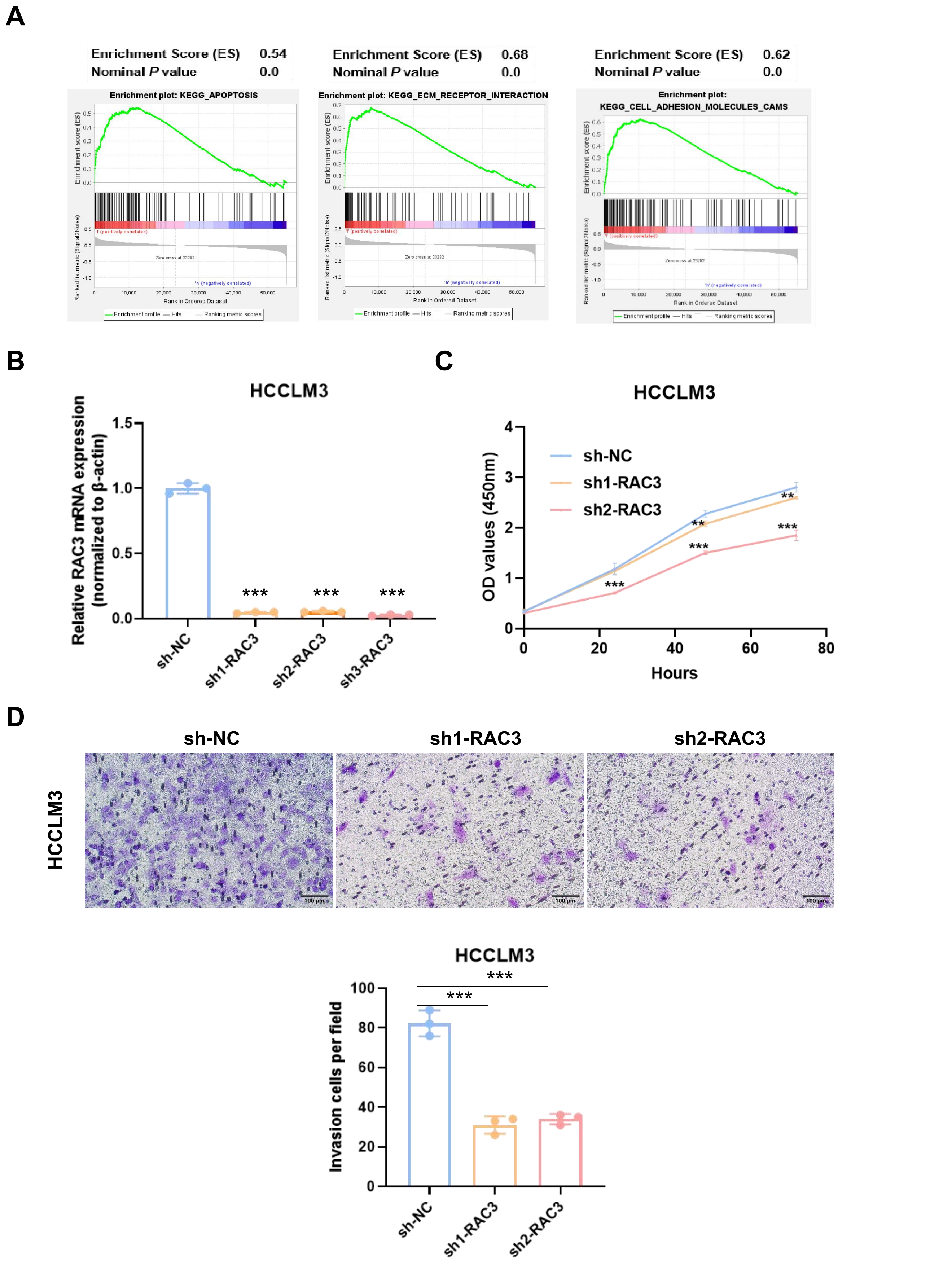
A
Enrichment Score (ES) 0.54
Nominal P value 0.0
Enrichment plot: KEGG_APOPTOSIS
Enrichment Score (ES) 0.68
Nominal P value 0.0
Enrichment plot: KEGG_ECM_RECEPTOR_INTERACTION
Enrichment Score (ES) 0.62
Nominal P value 0.0
Enrichment plot: KEGG_CELL_ADHESION_MOLECULES_CAMS
Enrichment score (ES)
Ranked list metric (Signal2Noise)
Zero cross at 23292
Rank in Ordered Dataset
Enrichment profile
Hits
Ranking metric scores
B
HCCLM3
Relative RAC3 mRNA expression (normalized to β-actin)
sh-NC
sh1-RAC3
sh2-RAC3
sh3-RAC3
***
C
HCCLM3
OD values (450nm)
Hours
sh-NC
sh1-RAC3
sh2-RAC3
**
***
D
sh-NC
sh1-RAC3
sh2-RAC3
HCCLM3
100 μm
HCCLM3
Invasion cells per field
***

Figure S7

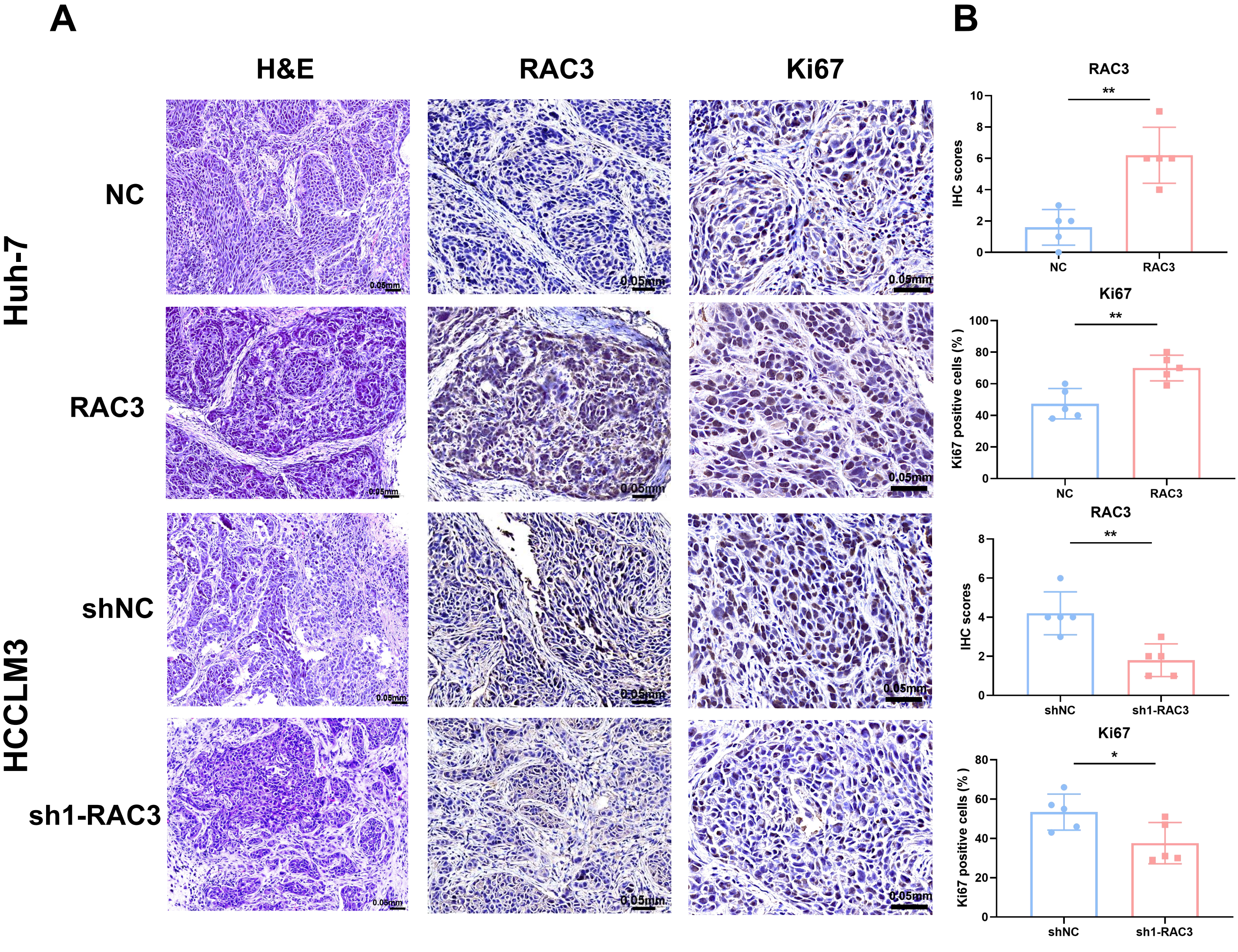
A
B
H&E
RAC3
Ki67
Huh-7
NC
RAC3
HCCLM3
shNC
sh1-RAC3
0.05mm
RAC3
IHC scores
NC
RAC3
**
Ki67
Ki67 positive cells (%)
NC
RAC3
**
RAC3
IHC scores
shNC
sh1-RAC3
**
Ki67
Ki67 positive cells (%)
shNC
sh1-RAC3
*

Figure S8

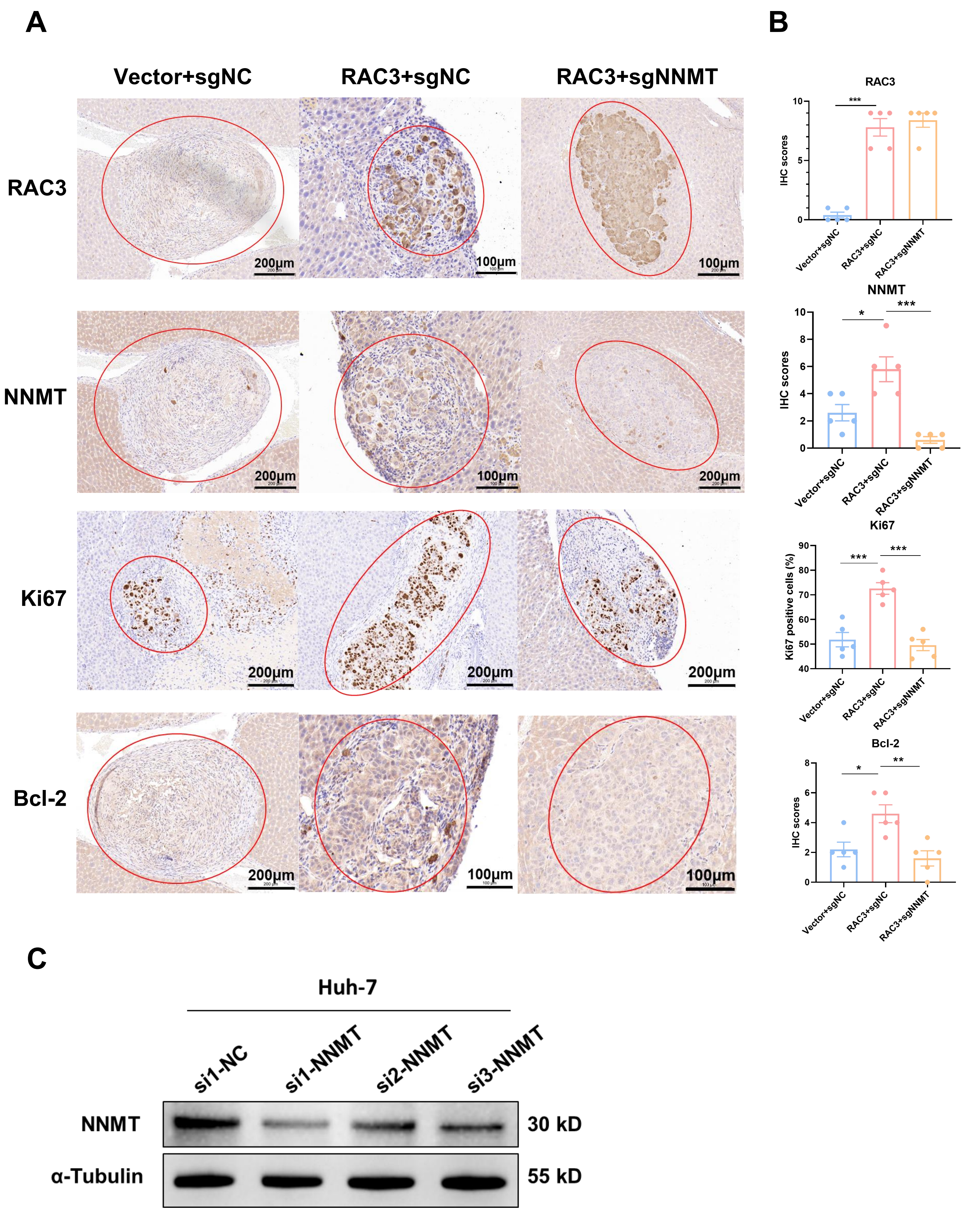
A
Vector+sgNC
RAC3+sgNC
RAC3+sgNNMT
RAC3
NNMT
Ki67
Bcl-2
200μm
100μm
B
RAC3
NNMT
Ki67
Bcl-2
IHC scores
Ki67 positive cells (%)
***
*
**
Vector+sgNC
RAC3+sgNC
RAC3+sgNNMT
C
Huh-7
si1-NC
si1-NNMT
si2-NNMT
si3-NNMT
NNMT
α-Tubulin
30 kD
55 kD

Figure S9

Download Graphics **Sort by:** E value

Sequence ID: Query_2371815 **Length: 2000** **Number of Matches: 3**

Range 1: 1655 to 1881 Graphics — Next Match | Previous Match

Score	Expect	Identities	Gaps	Strand
112 bits(123)	1e-27	170/237(72%)	19/237(8%)	Plus/Plus

Human / **Mouse**

```
Query  1555  TCCAGGCTTTTAAAATTACTCTTTCCAGACTTCATCTGAGA--CTCCTTCTTCAGCCAAC  1612
Sbjct  1655  TCCATGTTTTAAAAATTAATCTTTCCAGACTCCATCCGAGATTATTTTTCTCTAGCCAAC  1714

Query  1613  ATTCCTTAGCCCTGAATACATTTCCTATCCTCATCTTTCCCTTCttttttttCCTTTCTT  1672
Sbjct  1715  ATTCCTTAGCCATGAGCTCGTTTCCTTACCCCA-CATTCCCTTTTCTTTTTT--TTTGTT  1771

Query  1673  TTACATGTTTAAATTTAAACCATTCTTCGTGAC----CCCT---TTTCTTGGGAGATTCA  1725
Sbjct  1772  -----TGTTCAAACTGAAACCATTCGGTGGGACTCTTCCCTGACCCTCCAGGGAGAATCC  1826

Query  1726  TGGCAAGAACGAGAAGAATGATGGTGCTTGTTAGGGGATGTCCTGTCTCTCTGAACT  1782
Sbjct  1827  TTGCAAGAACAAGCAGACTACTG--GATTGTTTGGAGATGACCCGTCTCTCTGGACT  1881
```

Range 2: 1930 to 2000 Graphics — Next Match | Previous Match | First Match

Score	Expect	Identities	Gaps	Strand
62.6 bits(68)	6e-13	60/78(77%)	7/78(8%)	Plus/Plus

```
Query  1880  TGCATACAGCTCATGGCCCTCCCTCTACCATACCCTCCACCCCCGTTCGCCTAAGCTCCC  1939
Sbjct  1930  TGCATGGAGTTCATGGTCCTCCCCCTGCCA-------CTCCCCTCTTCTCCTAAGCTCCC  1982

Query  1940  TTCTCCGGGAATTTCATC  1957
Sbjct  1983  TTCTCCAGGAATTTCATC  2000
```

Range 3: 1066 to 1094 Graphics — Next Match | Previous Match | First Match

Score	Expect	Identities	Gaps	Strand
40.1 bits(43)	6e-06	26/29(90%)	0/29(0%)	Plus/Plus

```
Query  1360  ACTTTGAAAGAACTATCAGGATTTGGATT  1388
Sbjct  1066  ACTTTGAAAGAACTGTCAGGGTTTGAATT  1094
```

Figure S10

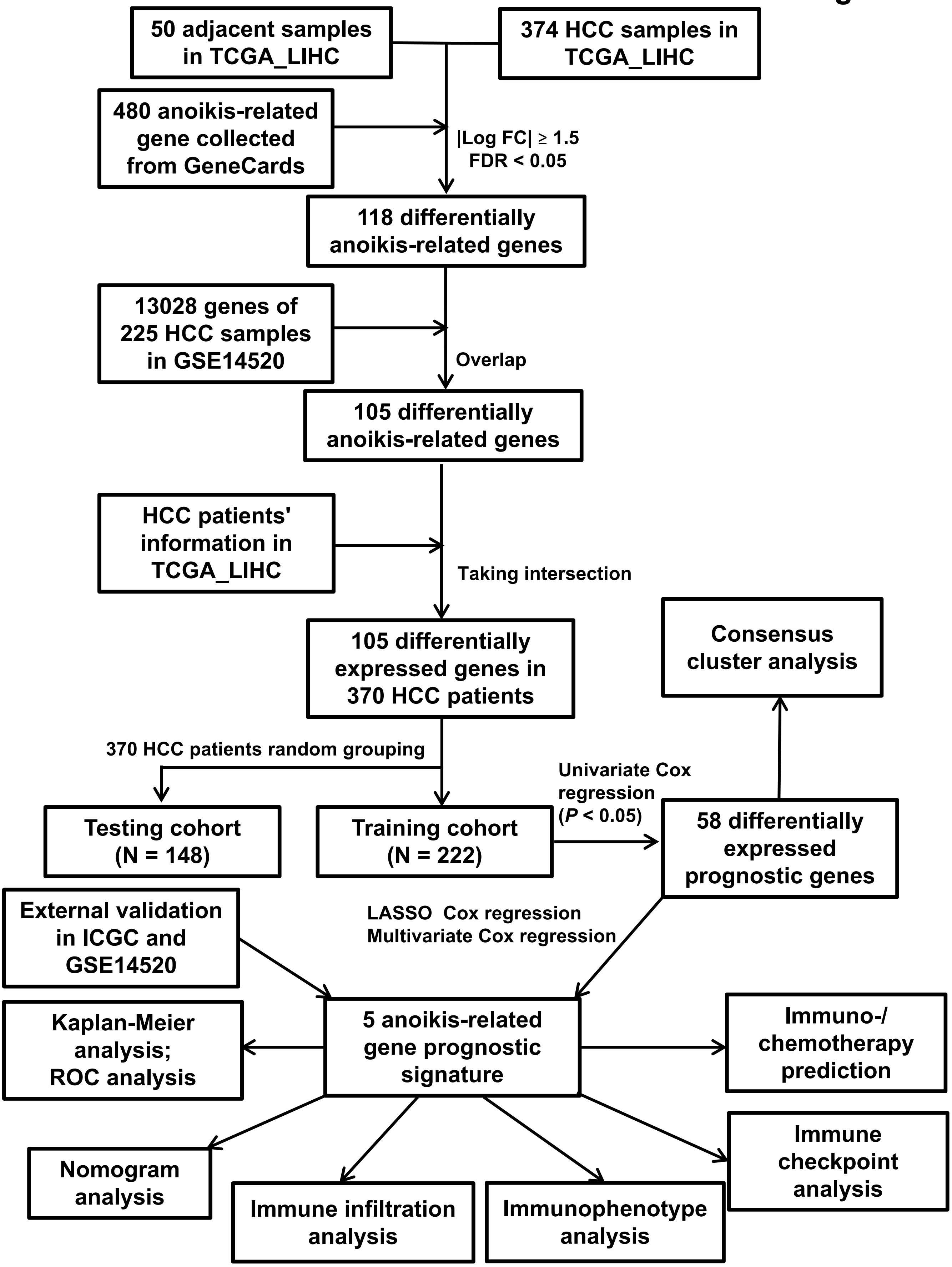
50 adjacent samples in TCGA_LIHC
374 HCC samples in TCGA_LIHC
480 anoikis-related gene collected from GeneCards
|Log FC| ≥ 1.5
FDR < 0.05
118 differentially anoikis-related genes
13028 genes of 225 HCC samples in GSE14520
Overlap
105 differentially anoikis-related genes
HCC patients' information in TCGA_LIHC
Taking intersection
105 differentially expressed genes in 370 HCC patients
Consensus cluster analysis
370 HCC patients random grouping
Univariate Cox regression (P < 0.05)
Testing cohort (N = 148)
Training cohort (N = 222)
58 differentially expressed prognostic genes
External validation in ICGC and GSE14520
LASSO Cox regression
Multivariate Cox regression
Kaplan-Meier analysis; ROC analysis
5 anoikis-related gene prognostic signature
Immuno-/ chemotherapy prediction
Nomogram analysis
Immune infiltration analysis
Immunophenotype analysis
Immune checkpoint analysis

Supplemental Figure Legends

Figure S1 Consensus clustering in HCC.

(A) Consensus matrix for HCC patients with $k = 2$. (B) Overall survival analysis of clusters C1 and C2 within the TCGA_LIHC cohort. (C) Transcriptional expression of 58 ARGs between clusters C1 and C2. (D) Top 15 up-regulated and 15 down-regulated DEGs between clusters C1 and C2. (E) KEGG analysis of DEGs. (F) GSEA of clusters C1 and C2 in TCGA_LIHC. Cluster C1: low ARG expression, Cluster C2: high ARG expression.

Figure S2 Nomogram and immune infiltration study.

(A) Nomograms for predicting the outcome of HCC patients, incorporating the ARG signature and clinicopathological factors based on TCGA_LIHC. (B) ROC analysis for the nomogram's 1-, 3-, and 5-year survival predictions. (C) Calibration plot assessing the nomogram's accuracy for predicting 1-, 3-, and 5-year overall survival. Analysis of immune cell (D) and immune function (E) scores within high-risk (N = 176) and low-risk (N = 184) subgroups. (F) Correlation between immune subtype classifications and risk stratification. Four distinct immune subtypes are identified: wound healing (Immune C1), interferon-γ dominant (Immune C2), inflammatory (Immune C3), and lymphocyte-depleted (Immune C4). (G) Comparative analysis of chemotherapy sensitivity in high-risk (N = 176) and low-risk (N = 184) patient groups. *$P < 0.05$, **$P < 0.01$, ***$P < 0.001$.

Figure S3 Expression and prognostic significance of the five ARGs in HCC.

(A) The mRNA expression levels of the five ARGs (SKP2, RAC3, SLC2A1, ETV4 and SPP1) within TCGA_LIHC cohort (Adjacent: N = 50, HCC: N = 374). (B) IHC staining of the five ARGs in HCC and adjacent normal tissues downloaded from the Human Protein Atlas. (C) Kaplan–Meier survival analysis comparing high versus low expression of the five ARGs in HCC patients. (D) Disease free survival analysis for patients with high or low expression of the five ARGs by GEPIA. ****P* < 0.001.

Figure S4 Expression and prognostic implications of RAC3 in pan-cancer.

(A) The mRNA expression of RAC1, RAC2 and RAC3 in TCGA_LIHC database (Adjacent: N = 50, HCC: N = 374). (B) The mRNA expression of RAC3 across cancers analyzed by TIMER. (C) Overall survival analysis for patients with high versus low RAC3 expression across six cancer types analyzed by Kaplan–Meier Plotter. **P* < 0.05, ***P* <0.01, ****P* < 0.001. (A) Mean ± SEM, Student's t test.

Figure S5 Correlation of RAC3 mRNA expression and prognosis in HCC with Stage II and AJCC_T2 by Kaplan-Meier Plotter.

Figure S6 Stable knockdown of RAC3 inhibits malignant phenotypes in HCC cells.

(A) GSEA showing the enrichment of genes related to apoptosis, ECM receptor interactions and cell adhesion molecules in HCC patients with high RAC3 expression

in the TCGA_LIHC dataset. The effect of siRNA knockdown of RAC3 and stable overexpression of RAC3 on the proliferation (B) qRT–PCR analysis of the expression of RAC3 in stable RAC3-knockdown HCCLM3 cells. The impact of stable RAC3 knockdown on the proliferation (C) and invasion (D) capabilities of HCC cells. $^{**}P < 0.01$, $^{***}P < 0.001$. Data represent mean ± SEM collected from three independent experiments.

Figure S7 RAC3 accelerates HCC tumorigenesis.

(A) Hematoxilin-eosin (H&E) staining and IHC analysis of the expression of RAC3 and Ki67 in xenograft tumors derived from human HCC cells (N = 5). (B) IHC scores associated with RAC3 and Ki67 expression (N = 5). $^{*}P < 0.05$, $^{**}P < 0.01$. Data represent mean ± SEM collected from three independent experiments.

Figure S8 RAC3 promotes orthotopic HCC proliferation mediated by NNMT. (A) IHC analysis of the expression of RAC3, NNMT, Ki67 and Bcl-2 in orthotopic liver tumors derived from MHCC97H cells (N = 5). (B) IHC scores associated with RAC3, NNMT, Ki67 and Bcl-2 expression (N = 5). (C) Western blot analysis to assess the knockdown efficiency of three siRNAs of NNMT. $^{*}P < 0.05$, $^{**}P < 0.01$. $^{***}P < 0.001$. Data represent mean ± SEM collected from three independent experiments.

Figure S9 DNA sequence alignment of human and mouse NNMT promoter region (2000bp) via NCBI blast.

Figure S10 Flowchart of the research program.

Supplemental Table S1 The clinical features of patients with HCC in testing and training cohorts

Covariates	Testing (N = 148)	Train (N = 222)	*P* value
**Age (years)**			0.5535
⩽ 65	96 (64.86%)	136 (61.26%)	
> 65	52 (35.14%)	86 (38.74%)	
**Gender**			0.8387
Female	47 (31.76%)	74 (33.33%)	
Male	101 (68.24%)	148 (66.67%)	
**Tumor grade**			0.7994
G1	23 (15.54%)	32 (14.41%)	
G2	72 (48.65%)	105 (47.30%)	
G3	45 (30.41%)	76 (34.23%)	
G4	6 (4.05%)	6 (2.70%)	
Unknow	2 (1.35%)	3 (1.35%)	
**Stage**			0.9733
Stage I	68 (45.95%)	103 (46.40%)	
Stage II	32 (21.62%)	53 (23.87%)	
Stage III	35 (23.65%)	50 (22.52%)	
Stage IV	2 (1.35%)	3 (1.35%)	
Unknow	11 (7.43%)	13 (5.86%)	

The 370 patients with HCC in TCGA_LIHC dataset were randomly divided into testing cohort and training cohort. Tumor grade is categorized as G1: well differentiated, G2: moderately differentiated, G3: poorly differentiated and G4: undifferentiated. Stage refers to the criteria from the American Joint Committee on Cancer (AJCC). *P* value is estimated by χ 2 test.

Supplemental Table S2. Correlation of RAC3 mRNA expression and prognosis in HCC with different clinicopathological factors by Kaplan-Meier Plotter.

Clinicopathological factors	Overall survival			Progression-free survival		
	N	Hazard ratio	P value	N	Hazard ratio	P value
**Gender**						
Female	118	1.52 (0.87-2.65)	0.13	120	1.23 (0.73-2.04)	0.43
Male	246	1.74 (0.99-3.07)	0.051	246	1.59 (1.07-2.23)	**0.02**
**Stage**						
I	170	1.45 (0.77-2.74)	0.25	170	0.59 (0.31-1.11)	0.099
II	83	2.81 (1.12-7.03)	**0.022**	84	2.31 (1.12-4.75)	**0.02**
I+II	253	1.74 (1.05-2.88)	**0.029**	254	1.43 (0.97-2.11)	0.066
III	83	0.71 (0.39-1.29)	0.26	83	1.58 (0.91-2.73)	0.1
IV	4	-	-	5	-	-
III+IV	87	0.74 (0.41-1.32)	0.31	88	1.49 (0.88-2.54)	0.14
**Grade**						
G1	55	0.55 (0.2-1.47)	0.23	55	2.17 (0.96-4.91)	0.056
G2	174	1.53 (0.89-2.62)	0.12	175	1.62 (1.03-2.54)	**0.035**
G3	118	3.11 (1.22-7.92)	**0.012**	119	1.7 (0.92-3.14)	0.085
G4	12	-	-	12	-	-
**AJCC_T**						
1	180	1.52 (0.83-2.8)	0.17	180	0.69 (0.38-1.24)	0.21
2	90	2.44 (1.08-5.52)	**0.027**	92	2.03 (1.07-3.86)	**0.028**
3	78	0.68 (0.37-1.25)	0.21	78	1.67 (0.95-2.95)	0.073
4	13	-	-	13	-	-

**Vascular invasion**						
Yes	90	0.67 (0.31-1.45)	0.3	91	1.67 (0.94-2.98)	0.076
None	203	0.45 (0.22-0.91)	**0.024**	204	0.67 (0.39-1.13)	0.13
**Race**						
White	181	1.43 (0.91-2.26)	0.12	183	0.72 (0.47-1.12)	0.14
Asian	155	1.62 (0.75-3.49)	0.21	155	1.99 (1.23-3.24)	**0.0044**
**Alcohol consumption**						
Yes	115	2.24 (1.06-4.74)	**0.031**	115	2.09 (1.15-3.83)	**0.014**
None	202	1.6 (0.89-2.88)	0.11	204	1.13 (0.92-2.06)	0.12
**Virus hepatitis**						
Yes	150	1.55 (0.73-3.3)	0.25	152	1.45 (0.91-2.3)	0.12
None	167	1.51 (0.96-2.38)	0.075	167	1.48 (0.93-2.35)	0.099

Tumor grade is categorized as G1:well differentiated, G2:moderately differentiated, G3:poorly differentiated and G4:undifferentiated. Stage refers to the criteria from the American Joint Committeeon Cancer(AJCC). *P* value is estimated by log-rank test.

Supplemental Table S3. Univariate analysis of clinicopathological factors for overall survival in patients with HCC

Clinicopathological features	HR (95% CI)[a]	*P* value
Age (≤ 60/> 60 years old)	0.848 (0.379-1.898)	0.688
Gender (male/female)	1.875 (0.447-7.862)	0.39
Tumor size (≥ 3 cm/< 3 cm)	5.865 (1.399-24.598)	**0.016**
Tumor number (1/> 1)	1.874 (0.765-4.590)	0.169
Tumor grade	1.544 (1.051-2.267)	**0.027**
Tumor capsule integrity (yes/no)	2.177 (1.017-4.662)	**0.045**
TNM stage	2.245 (1.257-4.011)	**0.006**
HBsAg (positive/negative)	0.932 (0.396-2.194)	0.873
AFP (≥ 25 μg/L/< 25 μg/L)	1.440 (0.685-3.029)	0.336
RAC3 expression (high/low)	4.013 (1.538-10.466)	**0.005**

HR, hazard ratio; CI, confidence interval.

Supplemental Table S4. Multivariate analysis of clinicopathological factors for overall survival in patients with HCC

Clinicopathological features	HR (95% CI)	*P* value
Tumor size (≥ 3 cm/< 3 cm)	4.659 (1.046-20.763)	**0.044**
Tumor grade (I-II/II/II-III/III)	1.114 (0.724-1.714)	0.625
Tumor capsule integrity (yes/no)	2.339 (1.024-5.340)	**0.044**
TNM stage (I, II, III)	1.552 (0.795-3.032)	0.198
RAC3 expression (high/low)	2.752 (1.026-7.376)	**0.044**

HR, hazard ratio; CI, confidence interval.

Supplemental Table S5. Interactive proteins with RAC3 identified by Co-IP/ MS

Accession	Description
Q9UJX6	Anaphase-promoting complex subunit 2 OS=Homo sapiens OX=9606 GN=ANAPC2 PE=1 SV=1
Q9BYZ6	Rho-related BTB domain-containing protein 2 OS=Homo sapiens OX=9606 GN=RHOBTB2 PE=1 SV=2
Q8N4E4	Phosducin-like protein 2 OS=Homo sapiens OX=9606 GN=PDCL2 PE=1 SV=2
P30740	Leukocyte elastase inhibitor OS=Homo sapiens OX=9606 GN=SERPINB1 PE=1 SV=1
Q9NU22	Midasin OS=Homo sapiens OX=9606 GN=MDN1 PE=1 SV=2
P35712	Transcription factor SOX-6 OS=Homo sapiens OX=9606 GN=SOX6 PE=1 SV=3
Q86UZ6	Zinc finger and BTB domain-containing protein 46 OS=Homo sapiens OX=9606 GN=ZBTB46 PE=1 SV=2
P50452	Serpin B8 OS=Homo sapiens OX=9606 GN=SERPINB8 PE=1 SV=2
Q8TE60	A disintegrin and metalloproteinase with thrombospondin motifs 18 OS=Homo sapiens OX=9606 GN=ADAMTS18 PE=1 SV=3
Q9UHK6	Alpha-methylacyl-CoA racemase OS=Homo sapiens OX=9606 GN=AMACR PE=1 SV=2
Q13129	Zinc finger protein Rlf OS=Homo sapiens OX=9606 GN=RLF PE=1 SV=2
Q13948	Protein CASP OS=Homo sapiens OX=9606 GN=CUX1 PE=1 SV=2
O60293	Zinc finger C3H1 domain-containing protein OS=Homo sapiens OX=9606 GN=ZFC3H1 PE=1 SV=3
Q8WYL5	Protein phosphatase Slingshot homolog 1 OS=Homo sapiens OX=9606 GN=SSH1 PE=1 SV=2
Q7Z5A9	Chemokine-like protein TAFA-1 OS=Homo sapiens OX=9606 GN=TAFA1 PE=1 SV=1
Q6N022	Teneurin-4 OS=Homo sapiens OX=9606 GN=TENM4 PE=1 SV=2
P20591	Interferon-induced GTP-binding protein Mx1 OS=Homo sapiens OX=9606 GN=MX1 PE=1 SV=4
Q68DK7	Male-specific lethal 1 homolog OS=Homo sapiens OX=9606 GN=MSL1 PE=1 SV=3
Q9NQ66	1-phosphatidylinositol 4,5-bisphosphate phosphodiesterase beta-1 OS=Homo sapiens OX=9606 GN=PLCB1 PE=1 SV=1
Q14764	Major vault protein OS=Homo sapiens OX=9606 GN=MVP PE=1 SV=4
Q2NL82	Pre-rRNA-processing protein TSR1 homolog OS=Homo sapiens OX=9606 GN=TSR1 PE=1 SV=1
Q8NFH8	RalBP1-associated Eps domain-containing protein 2 OS=Homo sapiens OX=9606 GN=REPS2 PE=1 SV=2
Q9NP74	Palmdelphin OS=Homo sapiens OX=9606 GN=PALMD PE=1 SV=1
P04003	C4b-binding protein alpha chain OS=Homo sapiens OX=9606 GN=C4BPA PE=1 SV=2
O14531	Dihydropyrimidinase-related protein 4 OS=Homo sapiens OX=9606 GN=DPYSL4 PE=1 SV=2
Q9BRX8	Peroxiredoxin-like 2A OS=Homo sapiens OX=9606 GN=PRXL2A PE=1 SV=3
Q9UPP2	IQ motif and SEC7 domain-containing protein 3 OS=Homo sapiens OX=9606 GN=IQSEC3 PE=2 SV=3
P55895	V(D)J recombination-activating protein 2 OS=Homo sapiens OX=9606 GN=RAG2 PE=1 SV=1
P41134	DNA-binding protein inhibitor ID-1 OS=Homo sapiens OX=9606 GN=ID1 PE=1 SV=3

Supplemental Table S6. Primer sequences

	Primers	Sequences
siRNAs for transfection	snc RNA-sense	5'-UUCUCCGAACGUGUCACGUTT-3'
	snc RNA-antisense	5'-ACGUGACACGUUCGGAGAATT-3'
	si1-RAC3-sense	5'-CCAAACUGACGUCUUUCUTT-3'
	si1-RAC3-antisense	5'-AGAAAGACGUCAGUUUGGGTT-3'
	si2-RAC3-sense	5'-CAAGGACACCAUUGAGCGGTT-3'
	si2-RAC3-antisense	5'-CCGCUCAAUGGUGUCCUUGTT-3'
	si1-NNMT-sense	5'-CUAUGUGUGUGAUCUUGAA(dT)(dT)-3'
	si1-NNMT-antisense	5'-UUCAAGAUCACACACAUAG(dT)(dT)-3'
	si2-NNMT-sense	5'-CGCUCAAGAGCAGCUACUA(dT)(dT)-3'
	si2-NNMT-antisense	5'-UAGUAGCUGCUCUUGAGCG(dT)(dT)-3'
	si3-NNMT-sense	5'-GCUCCUCUCUGCUUGUGAA(dT)(dT)-3'
	si3-NNMT-antisense	5'-UUCACAAGCAGAGAGGAGC(dT)(dT)-3'
For qRT-PCR	RAC3-F	5'-TCCCCACCGTTTTGACAACT-3'
	RAC3-R	5'-GCACGAACATTCTCGAAGGAG-3'
	β-actin-F	5'-AGCGAGCATCCCCCAAAGTT-3'
	β-actin-R	5'-GGGCACGAAGGCTCATCATT-3'
	BCL2-F	5'-GGTGGGGTCATGTGTGTGG-3'
	BCL2-R	5'-CGGTTCAGGTACTCAGTCATCC-3'
	SNAI1-F	5'-TCGGAAGCCTAACTACAGCGA-3'
	SNAI1-R	5'-AGATGAGCATTGGCAGCGAG-3'
	TWIST1-F	5'-GCCAGGTACATCGACTTCCTCT-3'
	TWIST1-R	5'-TCCATCCTCCAGACCGAGAAGG-3'
	NFKB1-F	5'-AACAGAGAGGATTTCGTTTCCG-3'
	NFKB1-R	5'-TTTGACCTGAGGGTAAGACTTCT-3'
	GBP1-F	5'-AGGAGTTCCTTCAAAGATGTGGA-3'
	GBP1-R	5'-GCAACTGGACCCTGTCGTT-3'
	NAT1-F	5'-TGGACTTAGGCTTAGAGGCCA-3'
	NAT1-R	5'-GGTCTCAAAACCAATAGTGGTCA-3'
	NNMT-F	5'- ATATTCTGCCTAGACGGTGTGA-3'
	NNMT-R	5'-TCAGTGACGACGATCTCCTTAAA-3'

BCL2: BCL2 apoptosis regulator, GBP1: Guanylate binding protein 1, NAT1: N-acetyltransferase 1, NFKB1: Nuclear factor kappa B subunit 1, NNMT: Nicotinamide N-methyltransferase, RAC3: Rac family small GTPase 3, SNAI1: Snail family transcriptional repressor 1, TWIST1: Twist family bHLH transcription factor 1
